# Changes of gut microbiota and short chain fatty acids in patients with Peutz–Jeghers syndrome

**DOI:** 10.1186/s12866-023-03132-0

**Published:** 2023-11-30

**Authors:** An Zhou, Bo Tang, Yuhong Xie, Shengpeng Li, Xu Xiao, Lingyi Wu, Dianji Tu, Sumin Wang, Yunxuan Feng, Xiaojie Feng, Yi Lai, Shoubin Ning, Shiming Yang

**Affiliations:** 1 Department of Gastroenterology, Second Affiliated Hospital, Army Medical University, Chongqing, 400037 China; 2 Department of Gastroenterology, Air Force Medical Center, Beijing, 100142 China

**Keywords:** Gut microbiota, Peutz–Jeghers syndrome, Short chain fatty acids, Benign polyps, Serine/threonine kinase 11

## Abstract

Peutz–Jeghers Syndromeis a rare autosomal dominant genetic disease characterized by gastrointestinal hamartomatous polyps and skin and mucous membrane pigmentation. The pathogenesis of PJS remains unclear; however, it may be associated with mutations in the STK11 gene, and there is currently no effective treatment available. The gut microbiota plays an important role in maintaining intestinal homeostasis in the human body, and an increasing number of studies have reported a relationship between gut microbiota and human health and disease. However, relatively few studies have been conducted on the gut microbiota characteristics of patients with PJS. In this study, we analyzed the characteristics of the gut microbiota of 79 patients with PJS using 16 S sequencing and measured the levels of short-chain fatty acids in the intestines. The results showed dysbiosis in the gut microbiota of patients with PJS, and decreased synthesis of short-chain fatty acids. *Bacteroides* was positively correlated with maximum polyp length, while *Agathobacter* was negatively correlated with age of onset. In addition, acetic acid, propionic acid, and butyric acid were positively correlated with the age of onset but negatively correlated with the number of polyps. Furthermore, the butyric acid level was negatively correlated with the frequency of endoscopic surgeries. In contrast, we compared the gut microbiota of STK11-positive and STK11-negative patients with PJS for the first time, but 16 S sequencing analysis revealed no significant differences. Finally, we established a random forest prediction model based on the gut microbiota characteristics of patients to provide a basis for the targeted diagnosis and treatment of PJS in the future.

**Supplementary Information:**

The online version contains supplementary material available at 10.1186/s12866-023-03132-0.

## Introduction

Peutz-Jeghers syndrome (PJS) is a rare, autosomal dominant genetic disorder characterized by mucocutaneous melanin deposits, gastrointestinal hamartomas, and an increased risk of gastrointestinal and extraintestinal cancers [1]. The estimated incidence of PJS is approximately 1 in 50,000 to 1 in 100,000 worldwide, and germline mutations of the tumor suppressor genes serine/threonine kinase 11 or liver kinase B 1 (STK11/LKB1) are considered to be the cause of PJS [2, 3]. STK11 is dependent on the phosphorylation and activation of the AMP-activated protein kinase (AMPK) for its actions. As a metabolic regulator, AMPK modulates metabolism by modulating the activity of metabolic enzymes and activating adaptive transcriptional responses, which may be related to the pathogenesis of PJS [4]. PJS-type hamartomatous polyps are most common in the gastrointestinal tract, predominantly in the colon and small intestine, but can also occur in extraintestinal sites [5]. In addition, hamartomatous polyps in patients with PJS can result in diverse gastrointestinal complications including abdominal pain, anemia, chronic bleeding, intussusception, and intussusception [6]. These acute and chronic complications seriously decrease life expectancy and affect the patients’ quality of life. Therefore, surveillance regimens and advanced endoscopy are recommended to help identify PJS at an early stage to limit the risk of emergency laparotomy. Endoscopic polypectomy is a common intervention for PJS patients with [7].

The gut microbiota is a complex ecosystem and there are more than 1000 different species of gut microbiota in the gut, which leads it to be called the “forgotten organ” [8]. Recent developments in sequencing have made it possible to investigate the relationship between the gut microbiota and human health and disease [9]. The homeostasis of the gastrointestinal tract is regulated by the exquisite balance of the immune system, gut microbiota, and metabolites, and the composition of the gut microbiota is influenced by numerous factors, including diet, genetics, environment, lifestyle, and medications [10]. In contrast, dysbiosis of gut microbiota may promote the pathogenesis of some gastrointestinal disorders including irritable bowel syndrome (IBS), inflammatory bowel disease (IBD), colorectal adenoma (CRA), and colorectal carcinoma (CRC) [11–14]. However, the role of the gut microbiota in the pathogenesis of PJS is still unknown, and more studies are required to elucidate the structure and function of the gut microbiota in patients with PJS.

To elucidate the role of gut microbiota in PJS patients, 16 S rRNA gene sequencing was performed on a cohort of 79 patients with PJS, 75 patients with benign polyps (adenomatous polyps in their pathology), and 83 healthy controls. Targeted metabolomics was further performed for 20 patients with PJS and 20 healthy controls to investigate the alteration of short-chain fatty acids (SCFAs) in patients with PJS. All of the subjects have been examined by endoscopy of the stomach, small intestine, and large intestine once a year in our study. Measurement of polyps number and length in the stomach, small intestine, and large intestine were included.

## Results

### Study population

A total of 79 patients with PJS (average age, 25.06 ± 11.82; ratio of males to females, 41:38; BMI, 21.03 ± 1.75), patients with benign polyps (average age, 48.03 ± 11.02; ratio of males to females, 35:40; BMI, 23.71 ± 3.14), and 83 healthy controls (average age, 27.64 ± 8.41; ratio of males to females, 46:37; BMI, 20.42 ± 2.08) were included in the analysis. The demographic and clinical characteristics were shown in Table 1. A total of 39 patients (49%) had experienced at least one intussusception, and 44 of these 79 patients (56%) had at least one personal history of endoscopic surgeries. A total of 28 patients (35%) had a family history, and 2 of these 79 cases (2.5%) were diagnosed with a tumor. In addition, other clinical characteristics (age of onset, 14.00 [8.00 ~ 20.00]; frequency of endoscopic surgeries, 2.50 [1.80 ~ 4.00]; length of the biggest polyps, 10 [4 ~ 23]; frequency of endoscopic surgeries, 1 [0 ~ 1]) were analyzed in Table 1.

**Table 1:** The demographics of patients. BMI, body mass index; Mutation, mutation of STK11 gene; Lenth, the lenth of biggest polyps; Polyps number, number of the PJS polyps; Surgery frequency, frequency of endoscopic surgeries; SD, standard deviation; IQR, interquartile range

Characteristic	PJS (*n* = 79)	Polyps (*n* = 75)	Control (*n* = 83)
Age, years (mean ± SD)	25.06 ± 11.82	48.03 ± 11.02	27.64 ± 8.41
Gender (male/female)	41/38	35/40 23.71 ± 3.14	46/37 20.42 ± 2.08
BMI, kg/m^2^ (mean ± SD)	21.03 ± 1.75		
Mutation, *n* (%)	43 (54%)		
Endoscopic surgeries, *n* (%)	44 (56%)		
Intussusception/obstruction, *n* (%)	39 (49%)		
Family history of PJS, *n* (%)	28 (35%)		
Cancer, *n* (%)	2 (2.5%)		
Age of onset, years (median [IQR])	14.00 (8.00 ~ 20.00)		
Lenth, cm (median [IQR])	2.50 (1.80 ~ 4.00)		
Polyps number, *n* (median [IQR])	10 (4 ~ 23)		
Surgery frequency, *n* (median [IQR])	1 (0 ~ 1)		

### Dysbiosis of gut microbiota in PJS patients compared with healthy controls

**Fig. 1:**
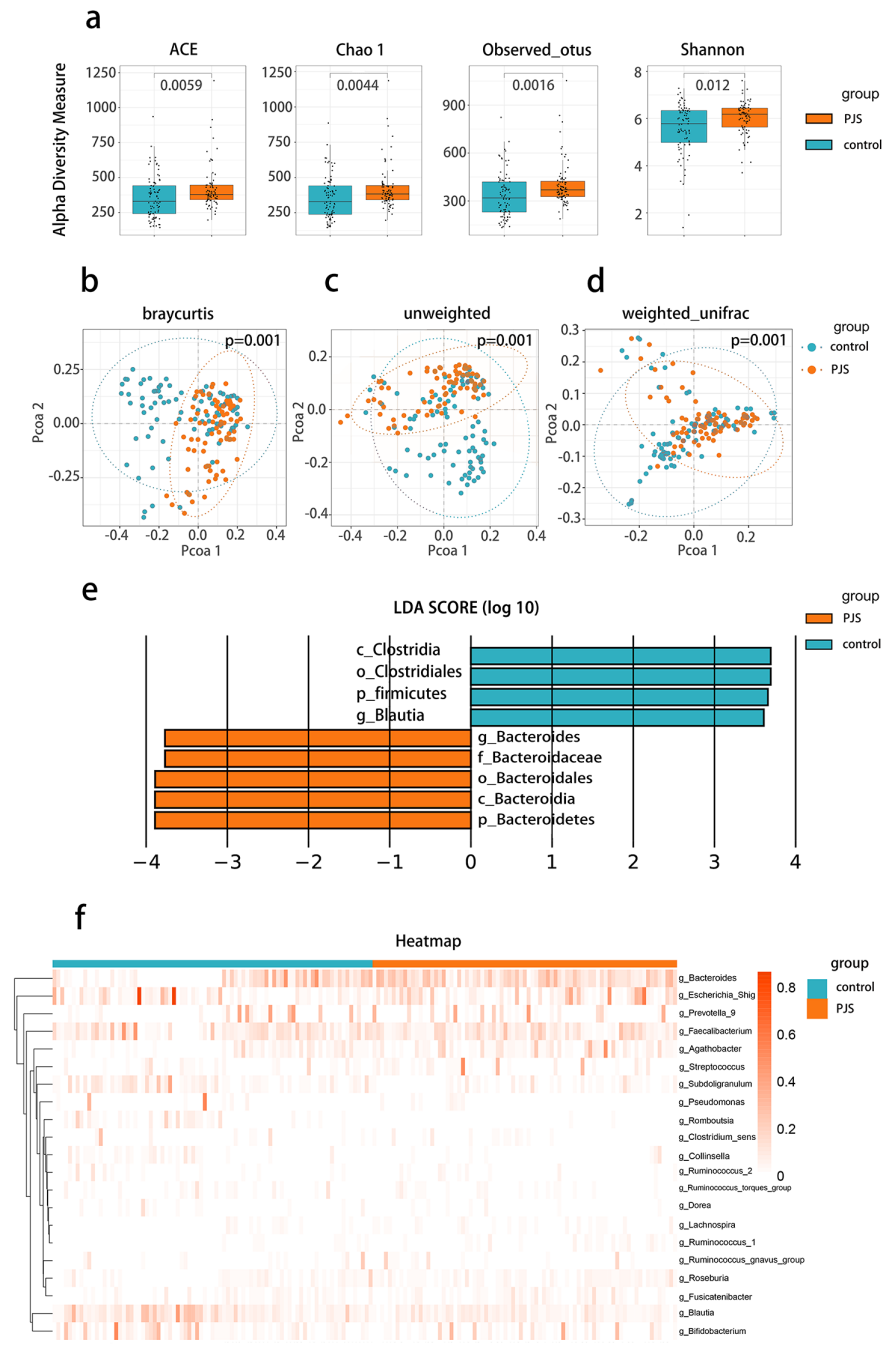
Dysbiosis of gut microbiota in PJS patients compared with healthy controls. **(a)** Alpha diversity boxplot (based on ACE, Chao1, observed otus and shannon). **(b-d)** Principal coordinate analysis (PCoA) using Bray-Curtis metric distances, Unweighted-UniFrac distance, and Weighted-UniFrac distance algorithms of beta diversity. **(e)** LEfSe analysis depicting taxonomic association between microbiome communities from PJS patients and healthy controls. **(f)** Heatmap of selected most differentially abundant features at the genus level

To characterize the signatures of gut microbiota in PJS patients, we collected stool samples from PJS patients and healthy controls and performed High throughput sequencing analysis. We originally measured gut microbial α-diversity with Wilcoxon signed-rank sum test. Consistently, different indices, including ace, chao1, observed_otus, and Shannon, showed similar tendencies and significant differences between PJS patients and healthy controls (*p* < 0.01, *p* < 0.01, *p* < 0.01, *p* < 0.05, Wilcoxon signed-rank sum test) (Fig. 1a). To identify the difference in the microbial community (β-diversity) between the two groups, principal coordinate analysis (PCoA) was performed based on Bray-Curtis metric distance, unweighted-UniFrac distance, and weighted-UniFrac distance algorithms (*p* = 0.001, *p* = 0.001, *p* = 0.001, PERMANOVA test) (Fig. 1b, c and d). An apparent clustering separation between amplicon sequence variants indicated different community structures between PJS patients and healthy controls. Subsequently, we assessed the features of gut microbiota from the phylum to genus levels to further evaluate the differences in the composition of gut microbiota between patients with PJS and healthy controls. At the phylum level of gut microbiota, the dominant taxa at the phylum level included *Firmicutes, Bacteroidetes, Proteobacteria*, and *Actinobacteria* (Figure. S1a). *Firmicutes* was the most predominant phylum, accounting for 62.99% and 57.22% of the gut microbiota in PJS patients and healthy controls, respectively (*p* = 0.019) (Figure S1b). Consistently, the abundance of *Bacteroidetes* was higher in the PJS group (*p* < 0.01), whereas that of *Actinobacteria* was higher in the control group (*p* < 0.01) (Figure S1c-1d). In addition, compositions of gut microbiota at the class, order, family, and genus levels were also compared between PJS patients and healthy controls (Figure S2a-S2d). At the genus level of gut microbiota, different biological compositions were observed between the two groups. In particular, the genera *Bacteroides, Agathobacter, Lachnospira, Roseburia*, and *Ruminococcus_1* were significantly enriched in the PJS group (*p* < 0.001, *p* < 0.001, *p* < 0.001, *p* < 0.001, and *p* < 0.001, respectively). In contrast, the genera *Subdoligranulum*, *Romboutsia, Blautia, Bifidobacterium*, and *Collinsella* were significantly enriched in the HC group of healthy controls (*p* < 0.001, *p* < 0.001, *p* < 0.001, *p* < 0.05, *p* < 0.05) (Figure S3).

To confirm which bacteria were associated with PJS, linear discriminant analysis (LDA) effect size (LEfSe) analysis was performed. LEfSe analysis showed eight discriminative features (LDA > 3.5; *p* < 0.05 [Fig. 1e]) at the genus (*n* = 2), family (*n* = 1), order (*n* = 2), class (*n* = 1), and phylum (*n* = 1) levels. The relative abundance of phylum *Bacteroidetes*, class *Bacteroidia*, order *Bacteroidales*, family *Bacteroidaceae*, and genus *Bacteroides* was higher in PJS patients, whereas the phylum *Firmicutes*, class *Clostridia*, order *Clostridiales*, and genus *Blautia* were significantly enriched in healthy controls. Furthermore, based on the abundance of gut microbiota at the genus level, we performed a comparison heatmap for the analysis of gut microbiota between the two groups, and the results were consistent with those shown in Figure S3 ​(Figure 1f). Collectively, the analysis indicated differences in the composition and structure of the gut microbiota in patients with PJS and healthy controls.

### Significant differences of gut microbiota between PJS patients and patients with benign polyps

**Fig. 2:**
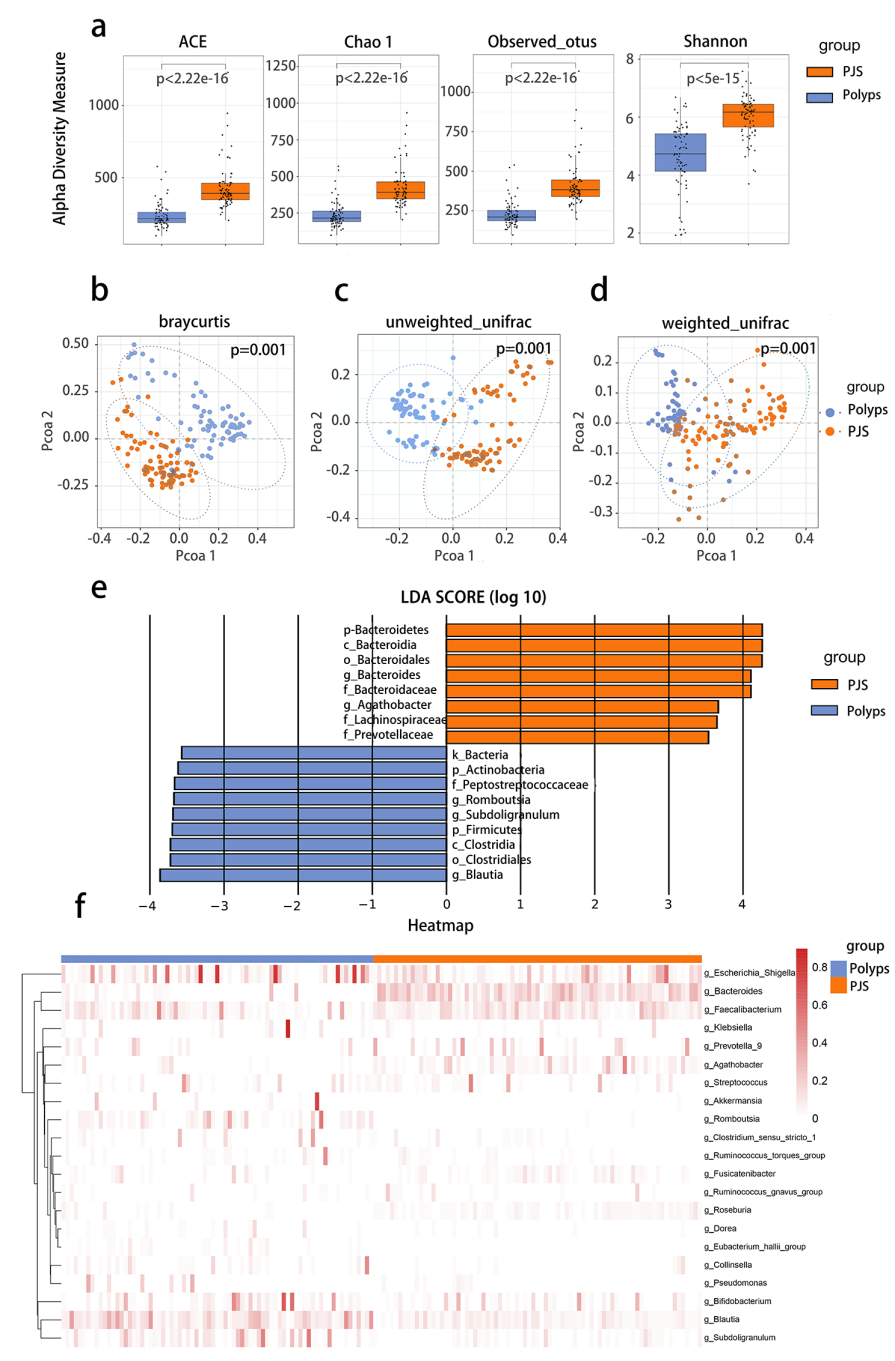
Difference of gut microbiota in PJS patients compared with patients with benign polyps. **(a)** Alpha diversity boxplot (based on ACE, Chao1, observed otus and shannon). **(b-d)** Principal coordinate analysis (PCoA) using Bray-Curtis metric distances, Unweighted-UniFrac distance, and Weighted-UniFrac distance algorithms of beta diversity. **(e)** LEfSe analysis depicting taxonomic association between microbiome communities from PJS patients and patients with benign polyps. **(f)** Heatmap of selected most differentially abundant features at the genus level

Gastrointestinal hamartomatous polyps are characteristic features of patients with Peutz-Jeghers syndrome. To identify the differences in gut microbiota between patients with PJS and patients with benign polyps, sequencing analysis of 16 S rRNA was performed. As for α-diversity, ace, chao1, observed otus, and shannon indices showed significant differences between the two groups (*p* < 0.001, *p* < 0.001, *p* < 0.001, *p* < 0.001, Wilcoxon signed-rank sum test) (Fig. 2a). In addition, β-diversity, including Bray-Curtis metric distance, unweighted-UniFrac distance, and weighted-UniFrac distance, showed a significant clustering separation between the two groups (*p* = 0.001, *p* = 0.001, *p* = 0.001, PERMANOVA test) (Fig. 2b-d), which revealed the different structure and composition of the gut microbiota in PJS patients and patients with benign polyps. However, the age of the benign polyps group is considerably higher than that of the PJS group, in that case, we conducted covariance analysis to adjust for the possible confounding factors (age, gender, and BMI) between two groups (Table S1). The results indicated that significant differences were still observed between two groups after adjusting for these characteristics. In addition, LEfSe analysis was conducted to confirm which bacteria were markedly enriched in PJS patients, and the results showed that the phylum *Bacteroidetes*, class *Bacteroidia*, order *Bacteroidales*, family *Bacteroidaceae*, family *Lachnospiraceae*, family *Prevotellaceae*, genus *Bacteroides*, and genus *Agathobacter* were enriched in PJS patients. In contrast, kindom *Bacteria*, phylum *Firmicutes*, phylum *Actinobacteria*, class *Clostridia*, order *Clostridiales*, family *Peptostreptococcaceae*, genus *Romboutsia*, genus *Subdoligranulum* and genus *Blautia* were enriched in patients with polyps (Fig. 2e). The differences in gut microbiota at the genus level between the two groups are shown in a heatmap (Fig. 2f), and the detailed features of gut microbiota at the phylum, class, order, family, and genus levels were also analyzed (Figure S4-S6).

### No significant difference of gut microbiota composition between STK11 positive and STK11 negative groups

**Fig. 3:**
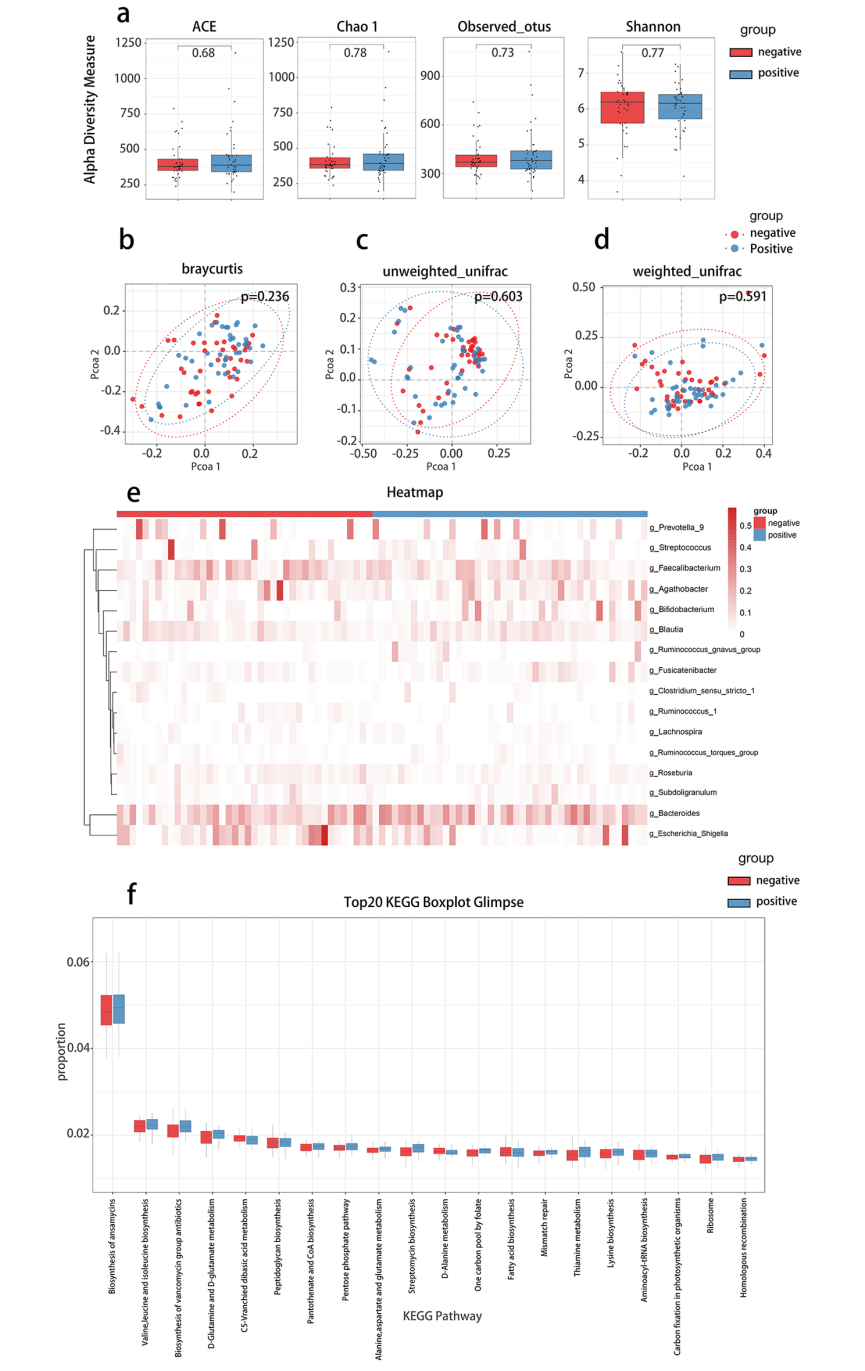
Difference of gut microbiota in STK11 positive patients compared with STK11 negative patients. **(a)** Alpha diversity boxplot (based on ACE, Chao1, observed otus and shannon). **(b-d)** Principal coordinate analysis (PCoA) using Bray-Curtis metric distances, Unweighted-UniFrac distance, and Weighted-UniFrac distance algorithms of beta diversity. **(e)** Heatmap of selected most differentially abundant features at the genus level. **(f)** Top20 KEGG Boxplot Glimpse

The STK11/LKB1 gene is involved in cell proliferation and cell-cycle signaling pathways, and the mutation of STK11 is considered to be the cause of PJS; 50–90% of PJS cases are due to mutations in this enzyme [15–17]. To identify whether the mutation of STK11 influences the structure and composition of gut microbiota, we compared the composition of gut microbiota in STK11 positive [43] and STK11 negative [36] patients with PJS. The α-diversity indices (ace, chao 1 indices, observed otus, shannon) (*p* = 0.68, *p* = 0.78, *p* = 0.73, *p* = 0.77, Wilcoxon signed-rank sum test) and β-diversity index (Braycurtis metric distance, unweighted-unifrac distance, weighted-unifrac distance) (*p* = 0.236, *p* = 0.603, *p* = 0.591, PERMANOVA test) showed no significant differences between the two groups (Fig. 3a and d). Subsequently, to identify the differences in microbial abundance between the two groups, we analyzed the composition of the gut microbiota at the phylum, class, order, family, and genus levels. At the phylum level, *Proteobacteria* were riched in STK11 negative group (*p* < 0.05) (Figure S7d). At the genus level, *Ruminococcus1* and *EscherichiaShigella* were riched in STK11 negative group (*p* < 0.05, *p* < 0.05), while *Bifidobacterium* was riched in STK11 positive group (*p* < 0.05) (Figure S9). The composition of the gut microbiota in each sample at the class, order, family, and genus levels are shown in Fig. 3e and Figure S8. The details of the gut microbiota composition at the genus level in each sample are shown in a heat map (Fig. 3e). In addition, we compared the expression of KEGG metabolic pathways between STK11 positive and negative patients. The results showed no significant differences in the expression of the top 20 metabolic pathways (Fig. 3f). According to these results, there was no significant difference in gut microbiota composition and abundance between the STK11 negative and positive groups, and the correlation between individual microbial community differences and genetic mutations still needs further validation.

### Synthesis of SCFAs decreased in PJS patients

**Fig. 4:**
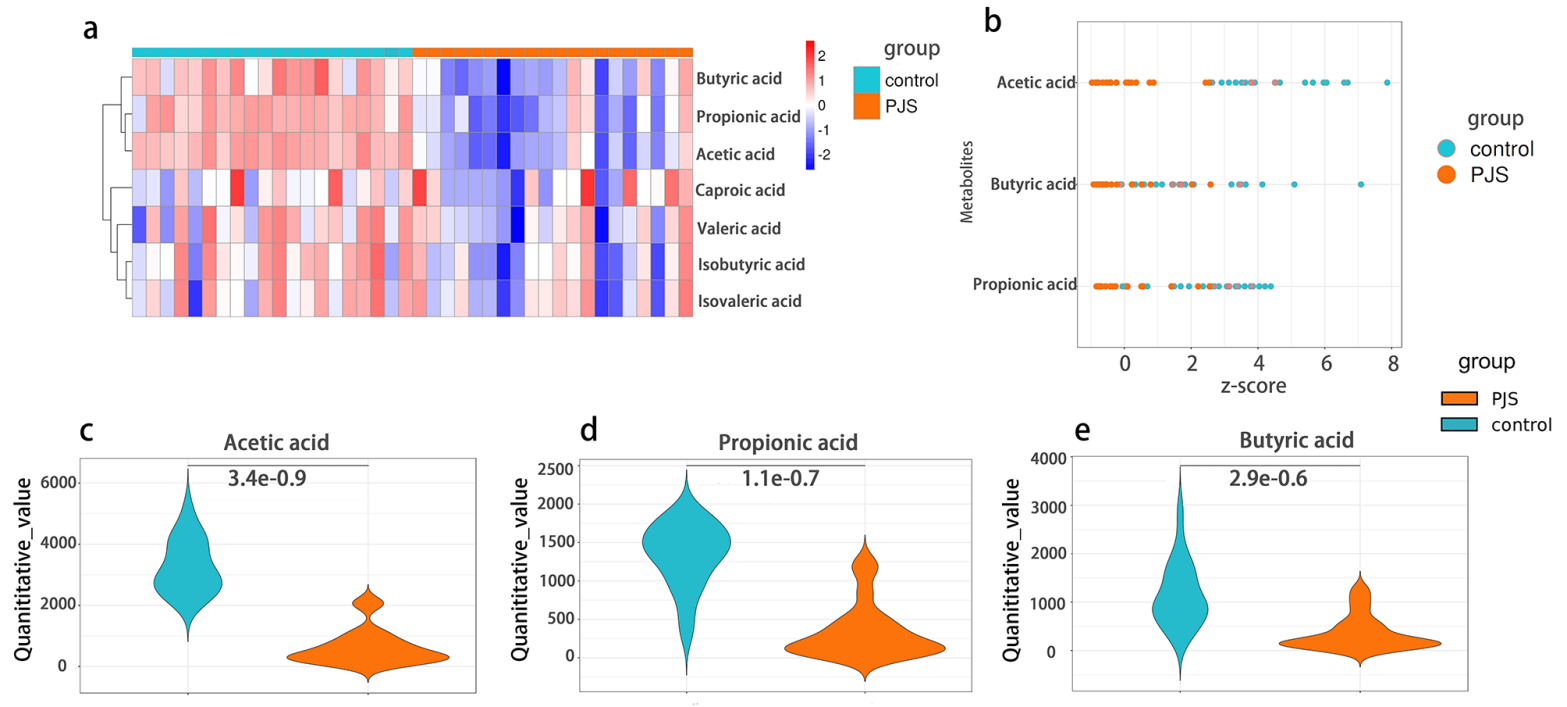
The synthesis of short-chain fatty acids is reduced in patients with PJS. **(a)** Heatmap of 7 kinds of SCFAs between PJS patients and healthy controls. **(b)** Z-score of three different SCFAs with variations. **(c-e)** Violin plot of acetic acid, propionic acid, and butyric acid between PJS patients and healthy controls

Previous 16 S rRNA sequencing analysis showed that *Subdoligranulum, Blautia*, and *Bifidobacterium* in the gut microbiota of PJS patients were decreased compared to healthy controls, which is associated with the metabolism of short-chain fatty acids (SCFAs) [18–20]. In addition, we used the Phylogenetic Investigation of Communities by Reconstruction of Unobserved States 2 (PICRUSt2) (V2.3.0) for functional prediction [21], and then mapped to the Kyoto Encyclopedia of Genes and Genomes (KEGG) pathway, the results indicated that expression of the fatty acid biosynthesis pathway was decreased in patients with PJS (Figure S10). In recent years, the importance of SCFAs in human health has been revealed, and SCFA are important fuels for intestinal epithelial cells (IEC) and regulate gut functions and host immunity [22]. In this case, in order to identify whether the metabolism of SCFA was altered in PJS patients, we performed a targeted metabolomics assay to detect 7 kinds of seven SCFAs in the feces of PJS patients and healthy controls, including acetic acid, propionic acid, isobutyric acid, butyric acid, isovaleric acid, valeric acid, and caproic acid. First, the content of seven types of SCFAs in each sample is shown in a total heatmap (Fig. 4a), and the SCFAs with significant differences were evaluated using the Z-score (Fig. 4b). The results showed that acetic acid, propionic acid, and butyric acid were enriched in healthy controls (*p* < 0.001, *p* < 0.001, and *p* < 0.001, respectively) (Fig. 4c and e), while the other four types of SCFAs showed no significant difference between the two groups. Therefore, we found that the synthesis of SCFAs was decreased in patients with PJS, which may influence the development of PJS.

**Fig. 5:**
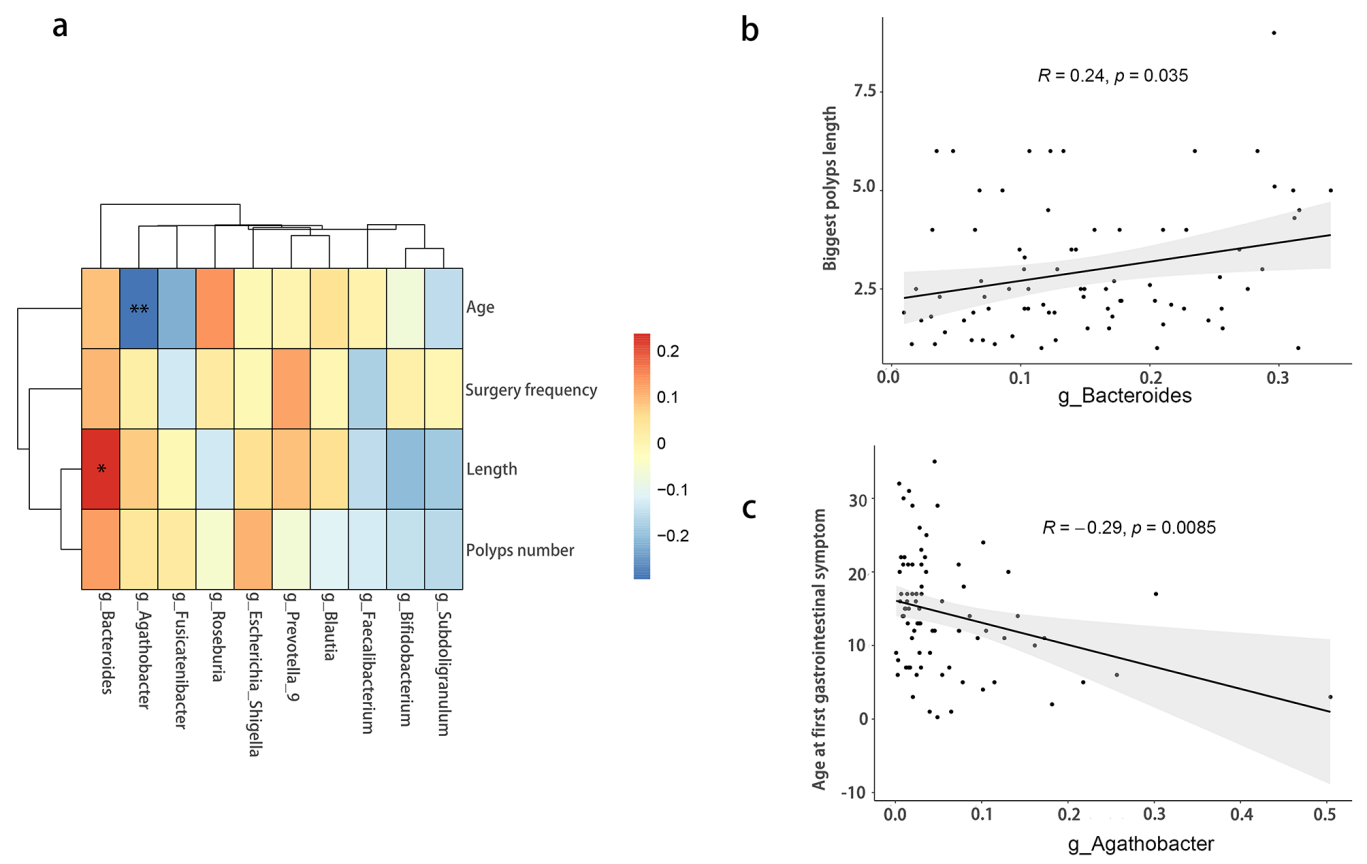
The correlation between gut microbiota and clinical features. **(a)** Heatmap of the correlation between selected gut microbiota at genus levels and clinical features. **(b-c)** Significant correlation between gut microbioat and clinical features

**Fig. 6:**
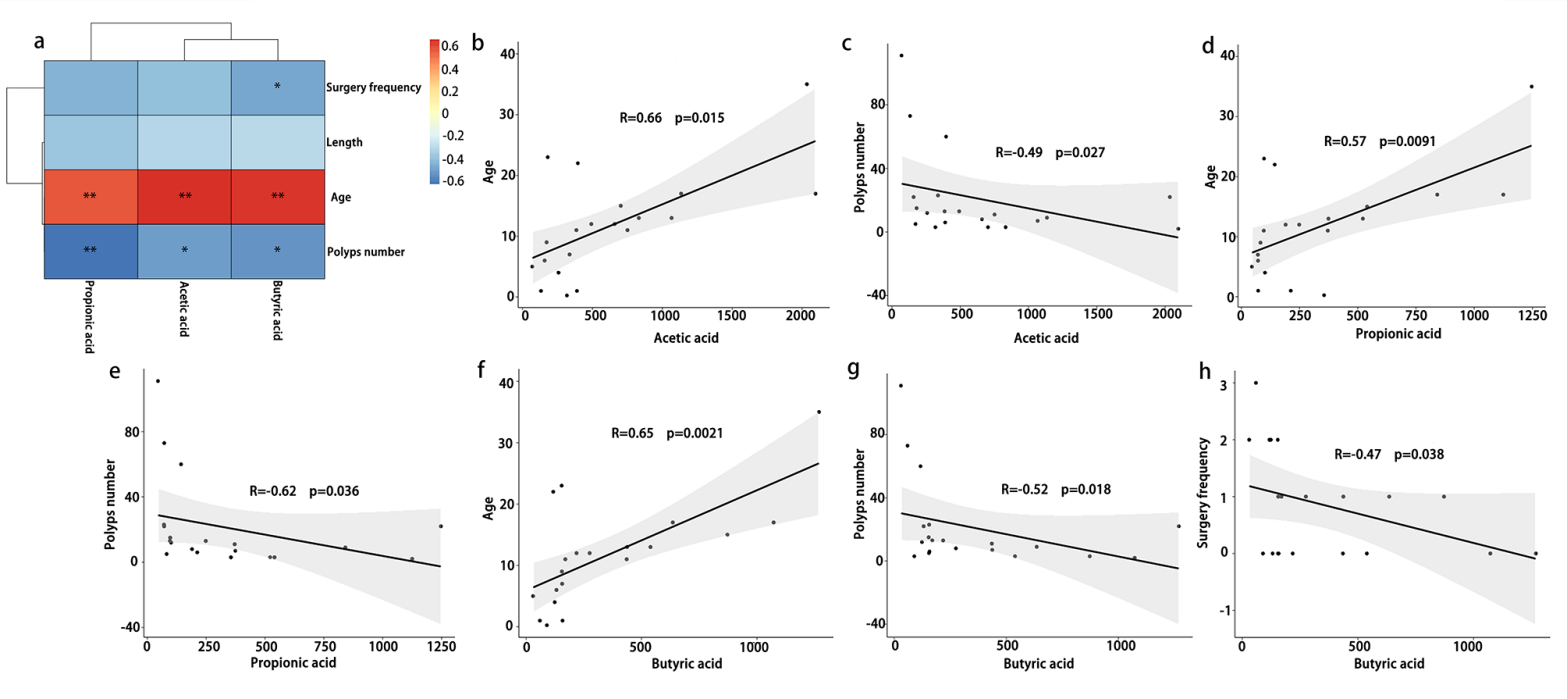
The correlation between SCFAs and clinical features. **(a)** Heatmap of the correlation between three types of SCFAs with significant differences and clinical features. **(b-h)** Significant correlation between SCFAs and clinical features (Age: age of onset. Surgery frequency: frequency of endoscopic surgeries. Length: the length of the biggest polyps. Polyps number: number of the PJS polyps)

**Fig. 7:**
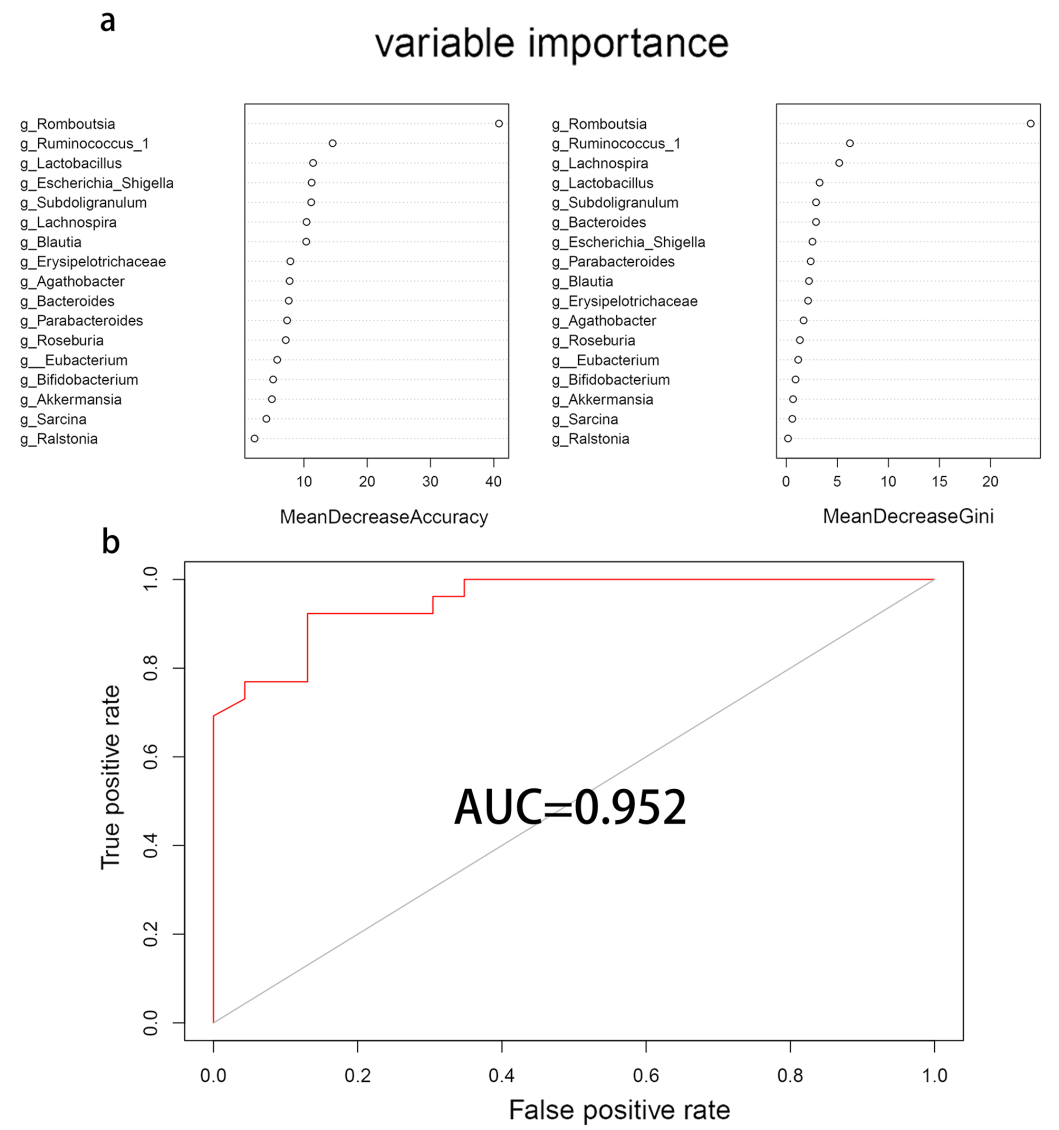
Random forest prediction model based on PJS patients. **(a)** Variable importance of the selected gut microbiota in genus level. **(b)** The Area Under the Curve of the random forest model

### *Bacteroides* is positively correlated with biggest polyps’ length while *Agathobacter* is negatively correlated with age at first gastrointestinal symptom

First, to identify the correlation between the gut microbiota and clinical characteristics in patients with PJS, a heatmap was drawn using Spearman’s correlation (Fig. 5a). The clinical features included age at first gastrointestinal symptom, frequency of endoscopic surgeries, largest polyp length, and number of PJS polyps. However, some gut microbiota with higher abundance at the genus level were selected, such as *Bacteroides, Agathobacter, Fusicatenibacter, Roseburia, Escherichia_Shigella, Prevotella_9, Blautia, Faecalibacterium, Bifidobacterium*, and *Subdoligranulum*. The analysis indicated that *Bacteroides* was positively correlated with the longest polyps’ length (r = 0.24, *p* = 0.035) (Fig. 5b), and *Agathobacter* was negatively correlated with age at first gastrointestinal symptoms (r = -0.29, *p* = 0.0085) (Fig. 5c). However, there was no significant correlation between the other gut microbiota and clinical features. These results indicate that *Bacteroides* may promote the growth of PJS polyps, and *Agathobacter* may be related to the onset of gastrointestinal symptoms in patients with PJS. However, further research is needed to demonstrate the influence of gut microbiota on the clinical characteristics of PJS patients.

### Higher content of acetic acid, propionic acid, and butyric acid are correlated with the improvement of clinical symptoms in PJS patients

In addition, to explore the relationship between SCFAs and clinical characteristics of PJS patients, we conducted a Spearman correlation analysis between the three SCFAs with significant differences (Fig. 6), including acetic acid, propionic acid, butyric acid, and frequency of endoscopic surgeries, maximum polyp length, age of onset, and number of polyps. The results showed that acetic acid, propionic acid, and butyric acid levels were positively correlated with the age of onset and negatively correlated with the number of polyps. In addition, butyric acid level was negatively correlated with surgical frequency in patients with PJS. Therefore, patients with higher levels of acetic acid, propionic acid, and butyric acid tend to have a later onset of initial symptoms and fewer polyps. Patients with higher levels of butyric acid underwent fewer endoscopic surgeries. The correlation of microbial feature and patient characteristics was summarized in Table S1.

### The occurrence of PJS could be predicted by the randomforest model of gut microbial signature

Recognizing that the features of gut microbiota may be a potential diagnostic biomarker for PJS, we established a random forest prediction model based on gut microbiota at the genus level to visualize specific taxa that contributed to the diagnostic potential between PJS and the control group. We used a training cohort containing 80% of patients with PJS (64/79) and a validation cohort containing 20% of patients with PJS (15/79). First, we set the seeds and drew a curve to describe the relationship between the number of decision trees and error rate. After variable importance screening, we selected the top Seventeen genus species, including *Romboutsia, Ruminococcus_1, Lactobacillus, Escherichia_Shigella, Subdoligranulum, Lachnospira, Blautia, Erysipelotrichaceae_UCG_003, Agathobacter, Bacteroides, Parabacteroides, Roseburia, Eubacterium_hallii_group, Bifidobacterium, Akkermansia, Sarcina*, and *Ralstonia*. The results of MeanDecreaseAccuracy and MeanDecreaseGini show the importance rank of each taxon (Fig. 7a). After training the model, its performance was verified in the validation cohort (AUC = 0.952; Fig. 7b).

## Discussion

PJS is a rare genetic disease characterized by gastrointestinal hamartomatous polyps and mucocutaneous melanin pigmentation, which lacks effective treatment options. In our study, we elucidated the features of the gut microbiota in PJS patients and found alterations in the gut microbiota of PJS patients compared to healthy controls or patients with benign polyps. For instance, phylum *Bacteroidetes* and genus *Bacteroides* were significantly enriched in PJS patients, while the abundance of phylum *Firmicutes*, phylum *Actinobacteria*, genus *Blautia*, and genus *Bifidobacterium* were decreased in PJS patients. *Bacteroides* is a major component of the gut microbiota that maintains intestinal homeostasis; nevertheless, *Bacteroides* could also play a pathogenic role in the host [23]. For instance, *Bacteroides vulgatus* has been found in patients with Crohn’s disease and may be involved in the development of this disease by disturbing the gut barrier or activating the inflammation response [24]. In addition, ectopic growth of *Bacteroides* can lead to diseases in other parts of the body, including meningitis, brain abscesses, and lung abscesses [25]. In addition, Melissa et al. found that *Bacteroides fragilis* is correlated with the level of inflammatory cytokines in the mucosa adjacent to the polyp and may induce a pro-inflammatory response by activating NF-κB through Toll-like receptor 4 and enriching the genes involved in LPS biosynthesis [26]. Furthermore, familial adenomatous polyposis (FAP), which is similar to PJS, was found to have enriched *Bacteroides fragilis* and *Bacteroides fragilis* toxin (bft) in the colonic mucosa compared to healthy individuals, which increased the risk of faster tumor onset and greater mortality of colorectal cancer (CRC) [27]. According to our results, *Bacteroides* were enriched in patients with PJS and positively correlated with the length of the largest polyps, which indicated that *Bacteroides* may exacerbate intestinal inflammation and promote the growth of PJ polyps. However, further research is required to elucidate the potential role of *Bacteroides* in the pathogenesis of PJS. On the other hand, some probiotics were reduced in patients with PJS compared with healthy controls, such as *Bifidobacterium* and *Blautia*, which play an important role in human health [28, 29]. Compared to the other studies, Wang et al. analyzed the composition of gut microbiota and fungus among 23 patients with PJS, 24 healthy controls, and 17 first-degree asymptomatic relatives by using 16 S and ITS2 sequencing. And they found that the composition and diversity of gut microbiota in patients with PJS were significantly different from those in healthy controls and asymptomatic relatives, with a lower frequency of the family *Ruminococcaceae*, family *Lachnospiraceae*, and the phylum *Firmicutes*, and a higher frequency of the genus *Escherichia-Shigella*, family *Enterobacteriaceae*, and phylum *Proteobacteria*. On the other hand, fungal flora was more stable [30]. Consistent with our results, Wang et al. indicated that genus *Bacteroides* were enriched, while the genus *Blautia* were decreased in patients with PJS. Besides, Du et al. investigated the feature of gut microbiota in 32 PJS patients and analyzed the relationship between gut microbiota and the growth of polyps [31]. The results suggested that the structure of the gut microbiota differed between healthy controls and PJS patients, while the richness was comparable. In addition, they found that *Morganella* was positively associated with the number of polyps (r = 0.96, *p* < 0.001), and *Desulfovibrio* was positively associated with number of newly discovered polyps in the jejunum between two recent endoscopic resections (r = 0.87, *p* = 0.01). Furthermore, Chen et al. indicated that the abundance of *Escherichia coli* increased, while the abundance of *Faecalibacterium prausnitzii* decreased in patients with PJS. And the reduction of *Faecalibacterium prausnitzii* was observed in patients with intussusception [32]. In addition, the age of onset may have an impact on the progression and treatment of the disease. For instance, previous studies suggested that patients with very early onset IBD (VEO-IBD) exhibit primary immune deficiencies (PIDs) and resistent to conventional therapy for IBD [33, 34]. And Yang et al. indicated that gut microbiota in patients with young-onset colorectal cancer exhibit a metabolic state that is more prone to malignant progression than patients with old-onset colorectal cancer, supporting the poorer prognosis of young-onset patients [35]. Based on these studies and our findings, we suggested that the gut microbiota and its metabolites may have a potential association with the age of onset in PJS patients, but currently there are few related reports. These findings may contribute to a better understanding of PJS. However, more clinical research is still needed.

In addition, the pathological features and severity of PJ-type polyps differ significantly from those of benign polyps. A PJ-type polyp is a hamartoma polyp that is commonly found in the small intestine. It is more prone to malignant transformation and can cause gastrointestinal bleeding, intestinal obstruction, and intussusception [36]. Therefore, we compared the composition of the gut microbiota between patients with PJS and patients with benign polyps. Similar to the above results, the α-diversity and β-diversity were significantly different between the two groups, and *Bacteroides* were enriched in PJS patients, which indicated a difference in gut microbial abundance and structure between PJS patients and patients with benign polyps. In addition, previous studies have indicated that human genetic variation is associated with an altered composition of the gut microbiome and leads to dysbiosis [37]. To investigate whether there are differences in gut microbiota between STK11 positive and STK11 negative patients with PJS, we performed 16s rRNA sequencing, which has not been previously reported. However, we found few differences between the two groups. After our analysis, due to the rarity of PJS, we only collected and analyzed the microbiota information of 79 patients, which may not be sufficient to solely focus on studying such a genetic disease. However, since the pathogenic mechanism of PJS is not yet fully understood, apart from mutations in the STK11 gene, other gene mutations may also be involved in the development of PJS syndrome [38]. In this case, more studies are required to elucidate the pathogenic mechanism of PJS, and the next step is to collect more PJS patients and their data on gut microbiota.

Based on the KEGG pathway analysis, the expression of the fatty acid biosynthesis pathway was decreased in PJS patients; therefore, we randomly selected 20 PJS patients and 20 healthy control fecal samples to measure the content of SCFAs and investigate whether the synthesis of SCFAs is decreased in the intestines of PJS patients. The results indicated that the contents of acetic acid, propionic acid, and butyric acid were significantly reduced in PJS patients, indicating that the synthesis of SCFAs decreased in PJS patients. Furthermore, through correlation analysis, we also found a positive correlation between SCFA levels and the clinical characteristics of patients, showing a positive effect. This suggests that interventions to supplement the levels of SCFAs in patients may help improve their clinical symptoms and prevent the occurrence of related complications, providing a new avenue for the treatment of PJS. However, further experimental and clinical validation are needed to confirm its effectiveness and safety. As previous studies reported, some gut microbiota, such as *Subdoligranulum*, *Blautia*, and *Bifidobacterium*, participate in the synthesis of SCFAs [18–20], and these gut microbiota decreased in PJS patients based on our study. Accumulating evidence has confirmed that SCFAs play an important role in the maintenance of gut and metabolic health, and SCFAs production is essential for gut integrity and mucosal immune function [39]. Therefore, dysbiosis of the gut microbiota and decreased synthesis of SCFAs may lead to destruction of the intestinal barrier and promotion of inflammatory responses.

Furthermore, we attempted to establish a random forest prediction model for PJS diagnosis based on the genus level of the gut microbiota, and 17 types of gut microbiota were selected. The results showed that the model is effective and feasible (AUC = 0.952), which provides a theoretical basis for the future diagnosis of PJS based on the gut microbiota. However, establishing the prediction model requires a larger sample size for the training and validation cohorts.

In summary, our research has found dysbiosis of gut microbiota in patients with PJS compared to healthy controls and patients with polyps, along with a decrease in the synthesis of short-chain fatty acids. Furthermore, we investigated whether there were differences in the gut microbiota between PJS patients positive and negative for the STK11 gene for the first time. Although the results showed no significant differences, further studies with larger sample sizes and consideration of other genes are required for validation. Finally, we developed a random forest prediction model based on the gut microbiota data of patients with PJS, providing a reference for future targeted modulation of the gut microbiota in the diagnosis and treatment of PJS. Due to the rarity and low incidence rate of PJS, our study only included 79 patients for analysis, which may have had an impact on the experimental results. This is also a limitation of our study. However, further research is needed to demonstrate the influence of gut microbiota on the clinical characteristics of PJS patients.

## Materials and methods

### Study population

All recruited patients with PJS were inpatients at the Second Affiliated Hospital of the Third Military Medical University, China. The research was approved by the Ethics Committee of the Second Affiliated Hospital of the Third Military Medical University. Individuals with (1) a positive family history of PJS and any number of histologically confirmed PJS polyps or characteristic, prominent, mucocutaneous pigmentation or (2) a negative family history of PJS and ≥ 3 histologically confirmed PJS polyps or any number of histologically confirmed PJS polyps and characteristic pigmentation were confirmed as having PJS [40].

Individuals were excluded if they had (1) received pharmacological agents, such as proton pump inhibitors (PPIs), antidepressants, antidiarrheal agents, laxatives, antibiotics, or probiotic supplements 4 weeks prior to the study; (2) had an enterectomy, an acute gastrointestinal hemorrhage, or intestinal neoplasia; or (3) were considered unsuitable for enteroscopy due to coagulopathy, severe cardiopulmonary diseases, or pregnancy. A total of 75 patients with benign polyps and 83 healthy controls were recruited for this study.

The methods for measuring the mutations of STK11 gene refered to our previous studies [41].

### Fecal sample collection and DNA extraction

Fecal samples were collected at Second Affiliated Hospital, the Third Military Medical University, delivered immediately to the laboratory at low temperatures, and then stored at − 80 °C until DNA extraction. DNA extraction from fecal samples was performed using a TIANamp Stool DNA Kit (Cat# DP328, TIANGEN Biotech Co. Ltd., China) according to the manufacturer’s instructions. A NanoDrop 2000 instrument (Thermo Scientific, Wilmington, NC, USA) was used to evaluate the concentration and purity of extracted DNA.

### 16s rRNA gene sequencing and quality control

The 16 S rRNA gene of the V3-V4 hypervariable regions (primers, 341 F: CCTAYGGGRBGCASCAG, 806R: GGACTACNNGGGTATCTAAT) was amplified to identify the diversity of gut microbiota. All PCR reactions were performed using 15µL of Phusion® High-Fidelity PCR Master Mix (New England Biolabs); about 10 ng template DNA, and 0.2 µM of forward and reverse primers. Thermal cycling consisted of initial denaturation for 1 min at 98℃, followed by 30 cycles of denaturation for 10s at 98℃, annealing for 30s at 50℃, and elongation at 72℃ for 30s and 72℃ for 5 min. For PCR product mixing and purification, mix the PCR product with an equal volume of 1X loading buffer (containing SYB Green) and perform electrophoresis on a 2% agarose gel. Mix the PCR product with an equal volume based on density ratio. Then, purify the mixed PCR product using a universal DNA purification kit (Tiangen, China, catalog number: DP214). And for library preparation and sequencing, the NEB Next® Ultra™ II FS DNA PCR-free Library Prep Kit (New England Biolabs, USA, Catalog #E7430L) was used to generate sequencing libraries, following the recommendations and indexes by manufacturers. The libraries were quantified using Qubit and real-time PCR, and their size distribution was analyzed using a bioanalyzer. Based on the effective library concentration and data amount required, the quantified libraries were pooled and sequenced on the Illumina platform (machine, Illumina novaseq 6000; sequencing type, 2 × 250 bp).

For paired-end reads assembly and quality control, paired-end reads were assigned to samples based on their unique barcodes and truncated by cutting off the barcode and primer sequence. For sequence assembly, paired-end reads were merged using FLASH (V1.2.11, http://ccb.jhu.edu/software/FLASH/) [42], which merges paired-end reads with overlapping ends from the same DNA segment, resulting in spliced sequences referred to as raw tags. And then, the fastp (Version 0.23.1) software was used to obtain high-quality clean tags for quality filtering on the raw tags [43]. After the above processing, the obtained tags need to undergo the process of removing chimeric sequences. The tags sequence is compared and checked for chimeric sequences with the Silva database, and the chimeric sequences are ultimately removed to obtain the final effective data (effective tags) [44]. To denoise the obtained effective tags mentioned above, DADA2 or deblur module in the QIIME2 software (Version QIIME2-202202) was performed to obtain initial amplicon sequence variants (ASVs) [45, 46].

In addition, species annotation, α-diversity, β-diversity were performed using QIIME2 software, and α-diversity indices were calculated with Wilcoxon signed-rank sum test, while β-diversity indices were calculated with PERMANOVA test. Besides, PCoA analysis was displayed by ade4 package (Version 1.7.15) [47] and ggplot2 package (Version 3.3.5) [48] in R software (Version 4.0.3), and heatmaps were achieved in R software through the pheatmap function. Furthermore, the predominance of gut microbiota between groups was analyzed by linear discriminant analysis (LDA) effect size (LEfSe) (LDA score (log10) = 3.5 as cutoff value) [49], and PICRUSt2 [21] was used for predicting functional abundances with Kyoto Encyclopedia of Genes and Genomes (KEGG) pathways based on marker gene sequences. On the other hand, throughout the analysis process, the version of Python used was 3.6.9.

### Metabolomics testing

Mixed standard stock solutions (100 mg/mL) of six SCFAs (acetic acid, propionic acid, isobutyric acid, butyric acid, isovaleric acid, and valeric acid) and a 100 mg/mL caproic acid stock solution was prepared using water and ether, respectively. Six SCFAs and caproic acid working solution series were prepared by appropriate dilutions of the standard stock solution. An internal standard (IS) solution (75 µg/mL) containing 4-methylvaleric acid was similarly prepared using ether. Ten points calibration curve was constructed by adding 110 µL of the working solutions containing 100 µL of six acid working solution series, 10 µL of caproic acid working solution series, 50 µL of 15%phosphoric acid, 10 µL of 75 µg/mL IS solution, and 130 µL of ether covering a range from0.02 to100 µg/mL (0.02, 0.1, 0.5, 1, 2, 5, 10, 25, 50, and 100 µg/mL). Stock solutions were stored at -20 °C before use, and working solutions were prepared for use. Samples were extracted in 50 µL of 15% phosphoric acid with 10 µL of 75 µg/mL 4-methylvaleric acid solution as the IS and 140 µL ether. Subsequently, the samples were centrifuged at 4 °C for 10 min at 12,000 rpm after vortexing for 1 min, and the supernatant was transferred into a vial rior to GC-MS analysis.

### Electronic supplementary material

Below is the link to the electronic supplementary material.

Supplementary Material 1

Supplementary Material 2

Supplementary Material 3

Supplementary Material 4

Supplementary Material 5

Supplementary Material 6

Supplementary Material 7

Supplementary Material 8

Supplementary Material 9

Supplementary Material 10

Supplementary Material 11

Supplementary Material 12

Supplementary Material 13

Supplementary Material 14

## Data Availability

The datasets generated and/or analysed during the current study are available in the Genome Sequence Archive repository for 16 S rRNA gene (CRA013560, https://ngdc.cncb.ac.cn/gsa/) and OMIX repository for targeted metabolomics assay of SCFAs (OMIX005264, https://ngdc.cncb.ac.cn/omix/).
