# Supplementary figures and images for: Changes of gut microbiota and short chain fatty acids in patients with Peutz–Jeghers syndrome
Source: BMC Microbiol. 2023 Nov 30;23:373. doi: 10.1186/s12866-023-03132-0 (PMC10688050; doi:10.1186/s12866-023-03132-0)

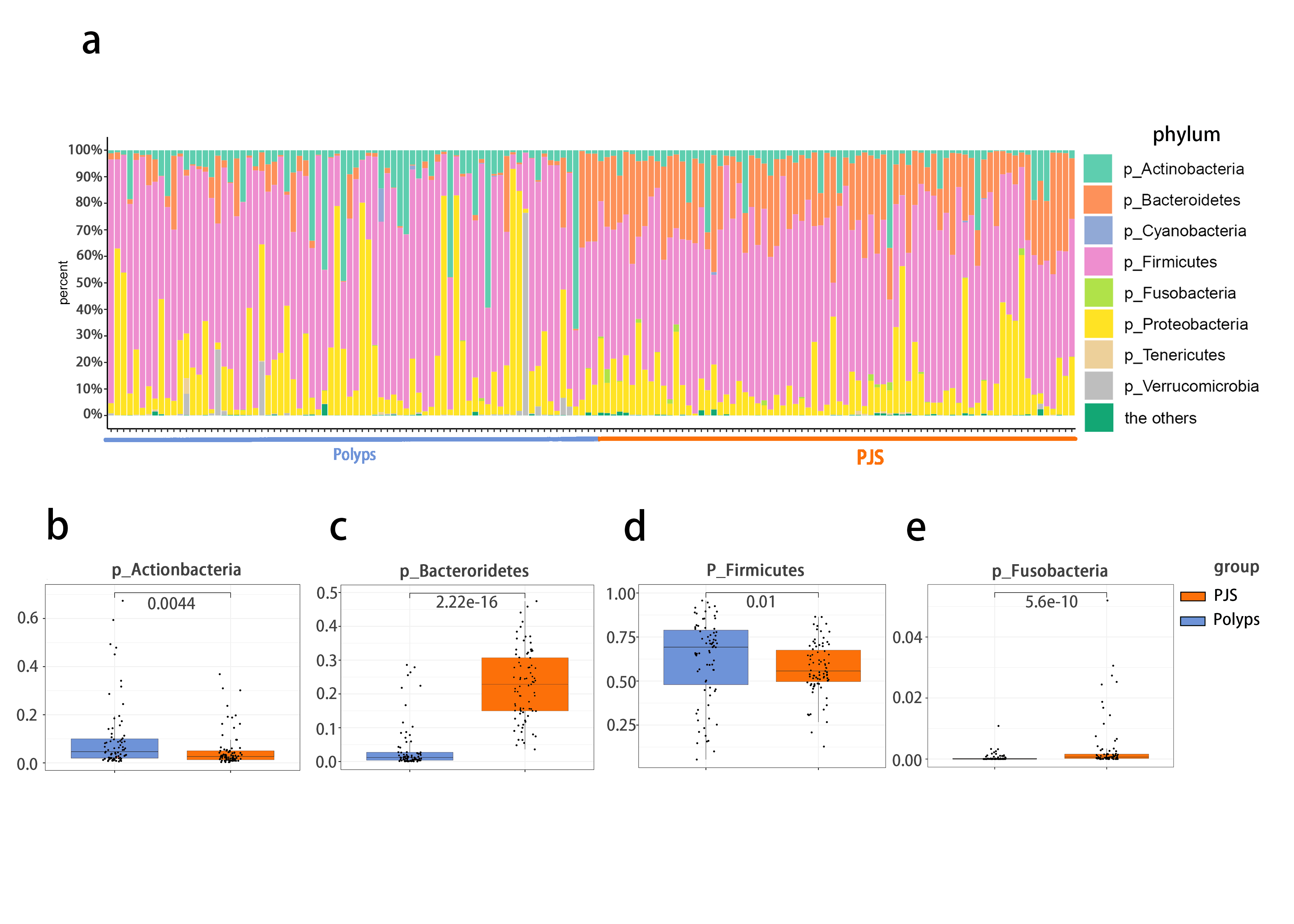

Supplement: Supplementary file 1 — Supplementary Material 1 [file 12866_2023_3132_MOESM1_ESM.png]

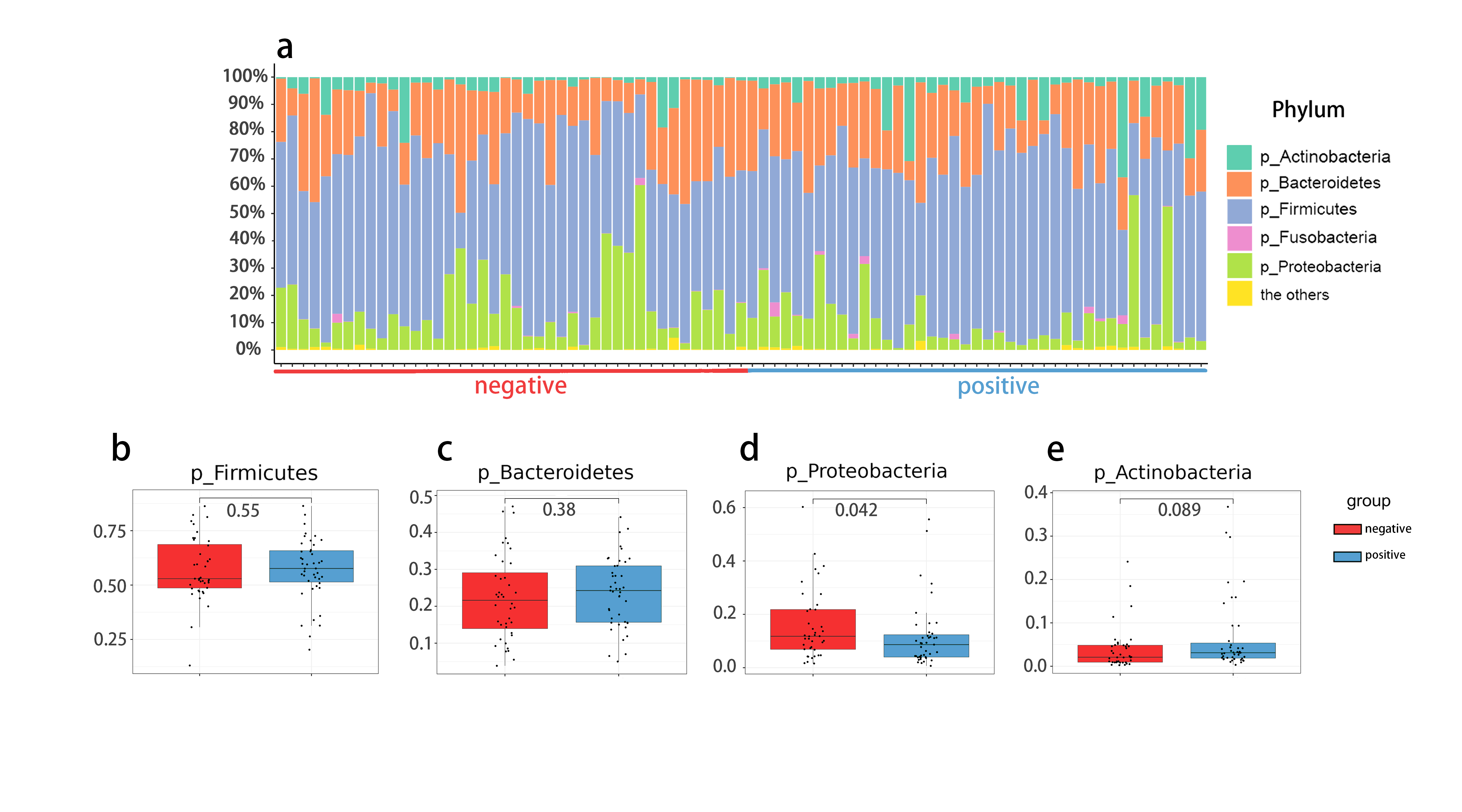

Supplement: Supplementary file 2 — Supplementary Material 2 [file 12866_2023_3132_MOESM2_ESM.png]

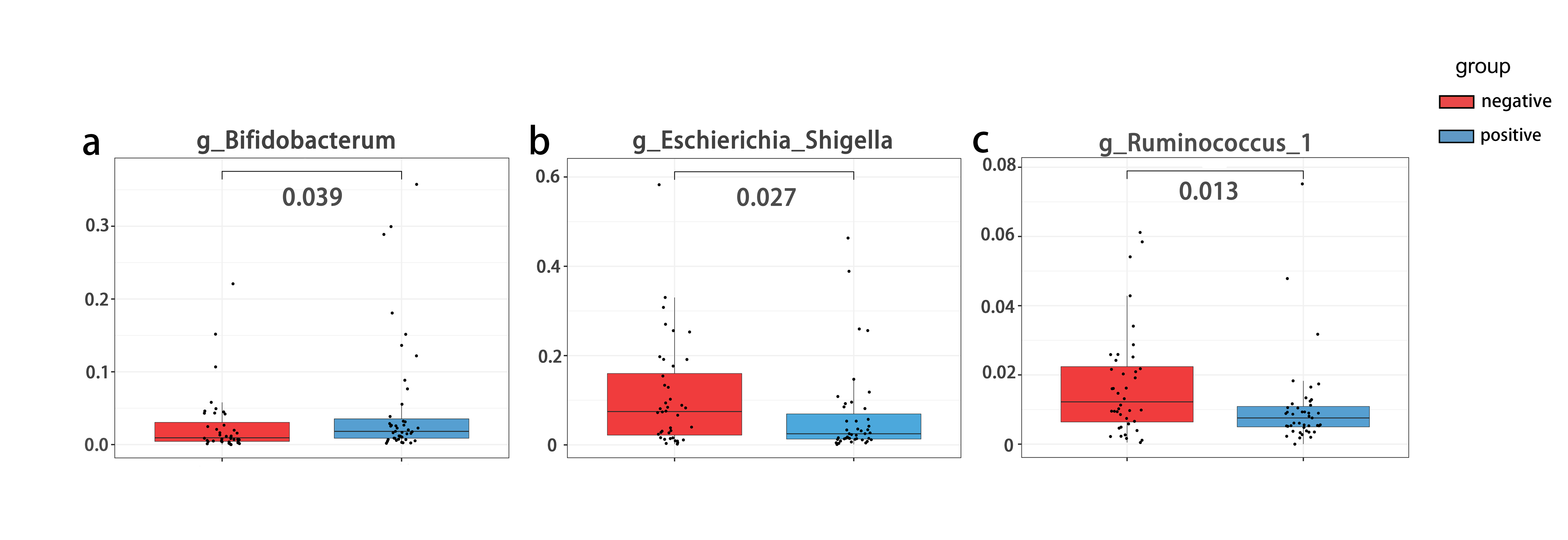

Supplement: Supplementary file 3 — Supplementary Material 3 [file 12866_2023_3132_MOESM3_ESM.png]

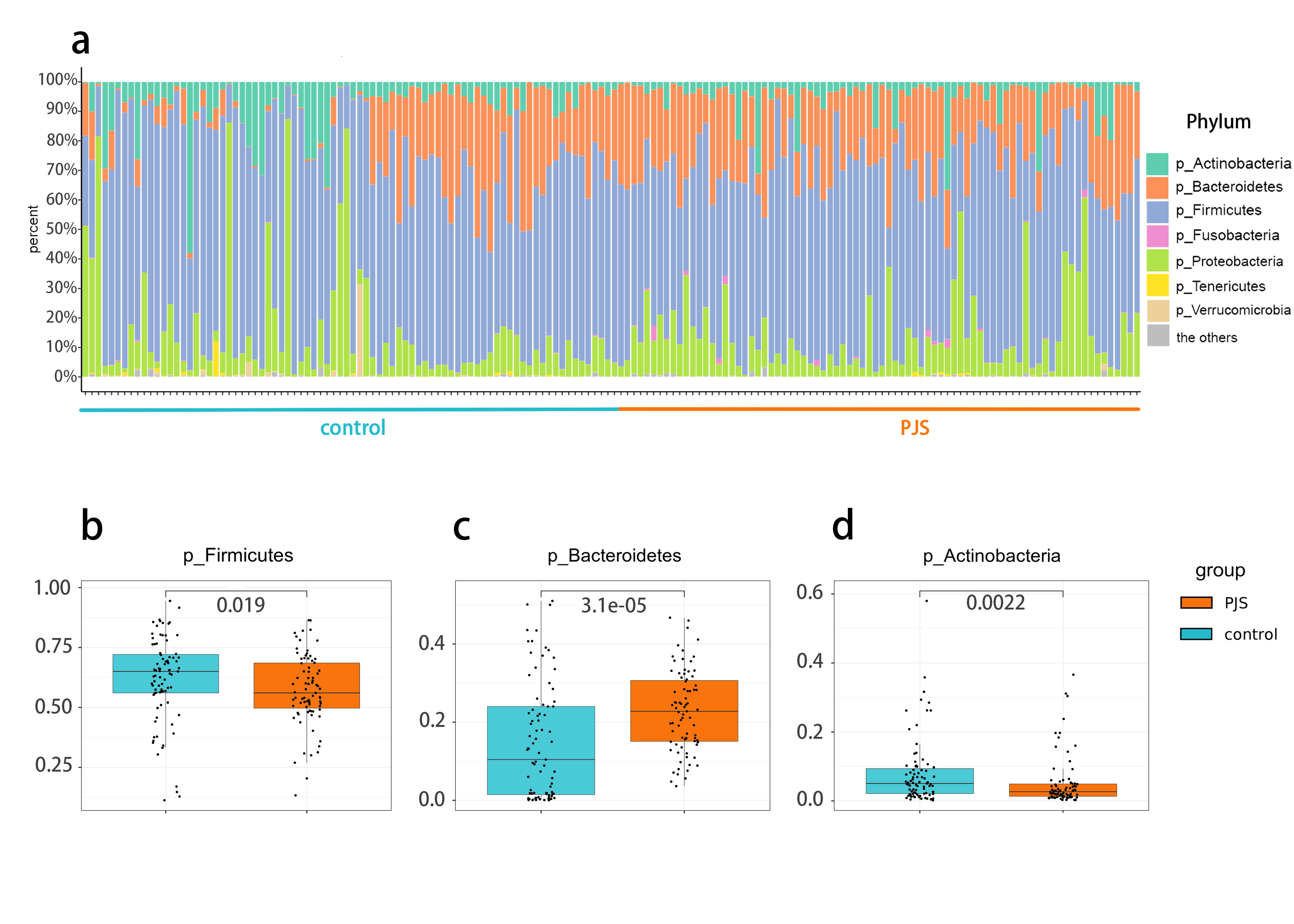

Supplement: Supplementary file 4 — Supplementary Material 4 [file 12866_2023_3132_MOESM4_ESM.png]

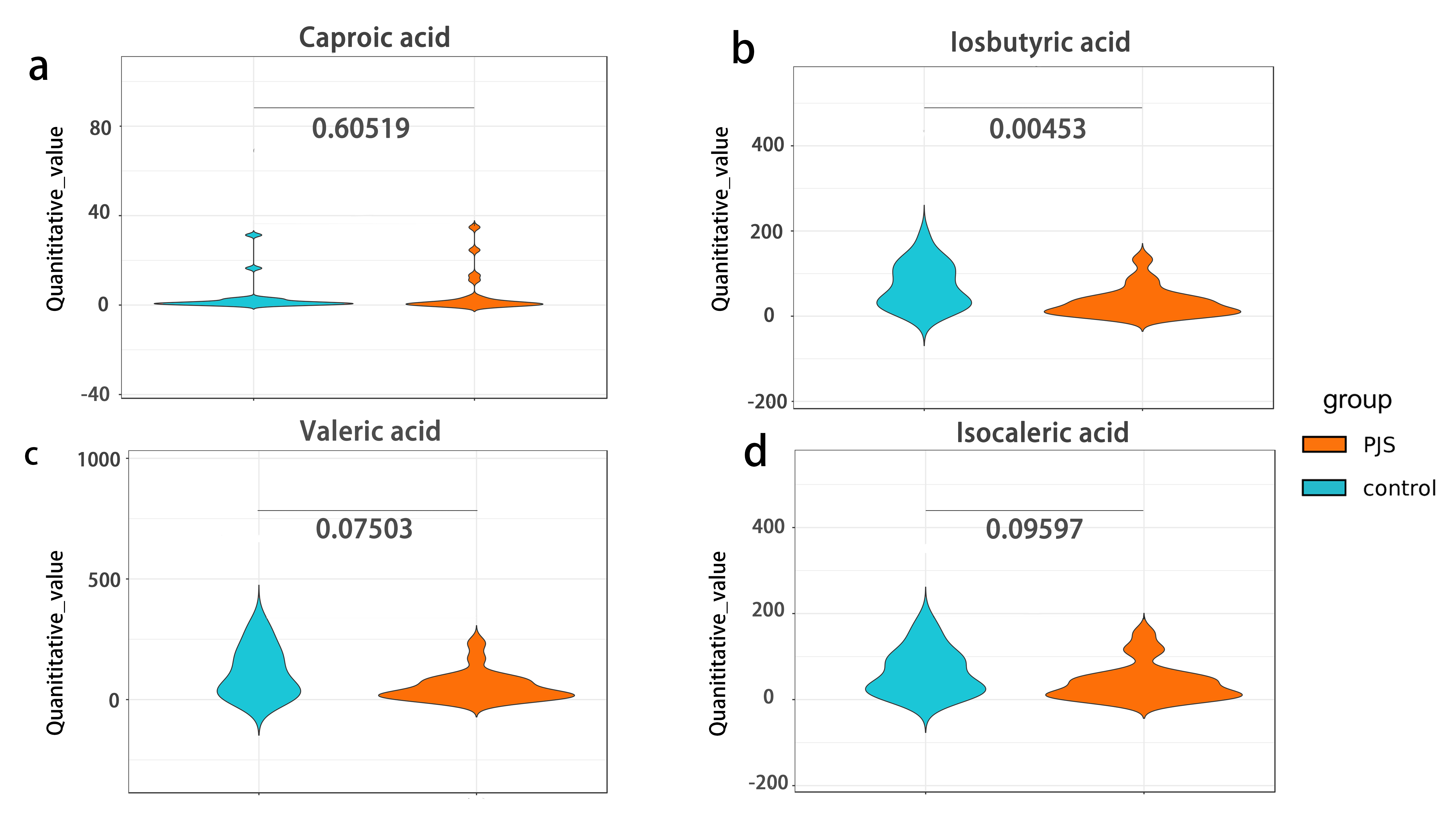

Supplement: Supplementary file 5 — Supplementary Material 5 [file 12866_2023_3132_MOESM5_ESM.png]

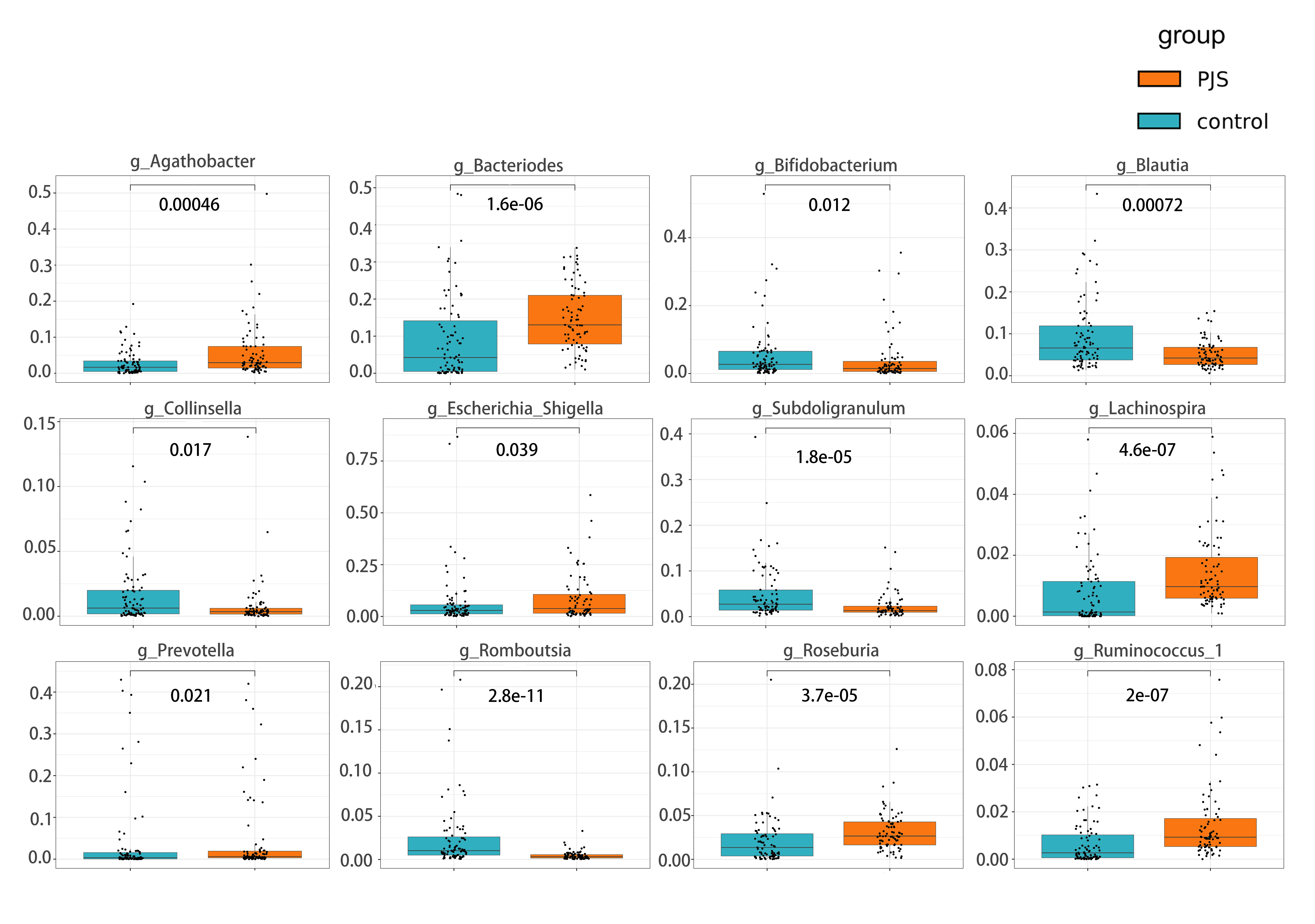

Supplement: Supplementary file 6 — Supplementary Material 6 [file 12866_2023_3132_MOESM6_ESM.png]

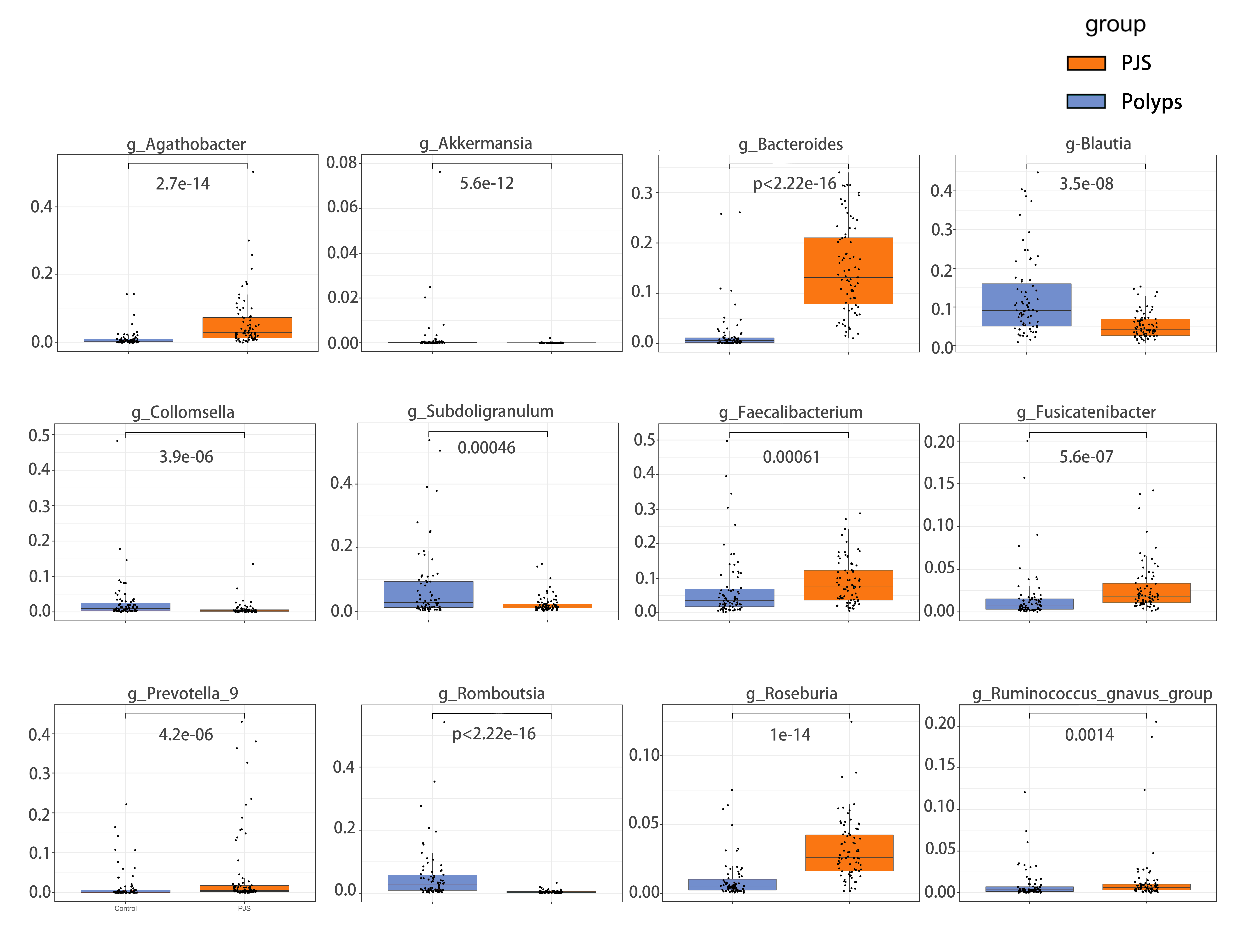

Supplement: Supplementary file 7 — Supplementary Material 7 [file 12866_2023_3132_MOESM7_ESM.png]

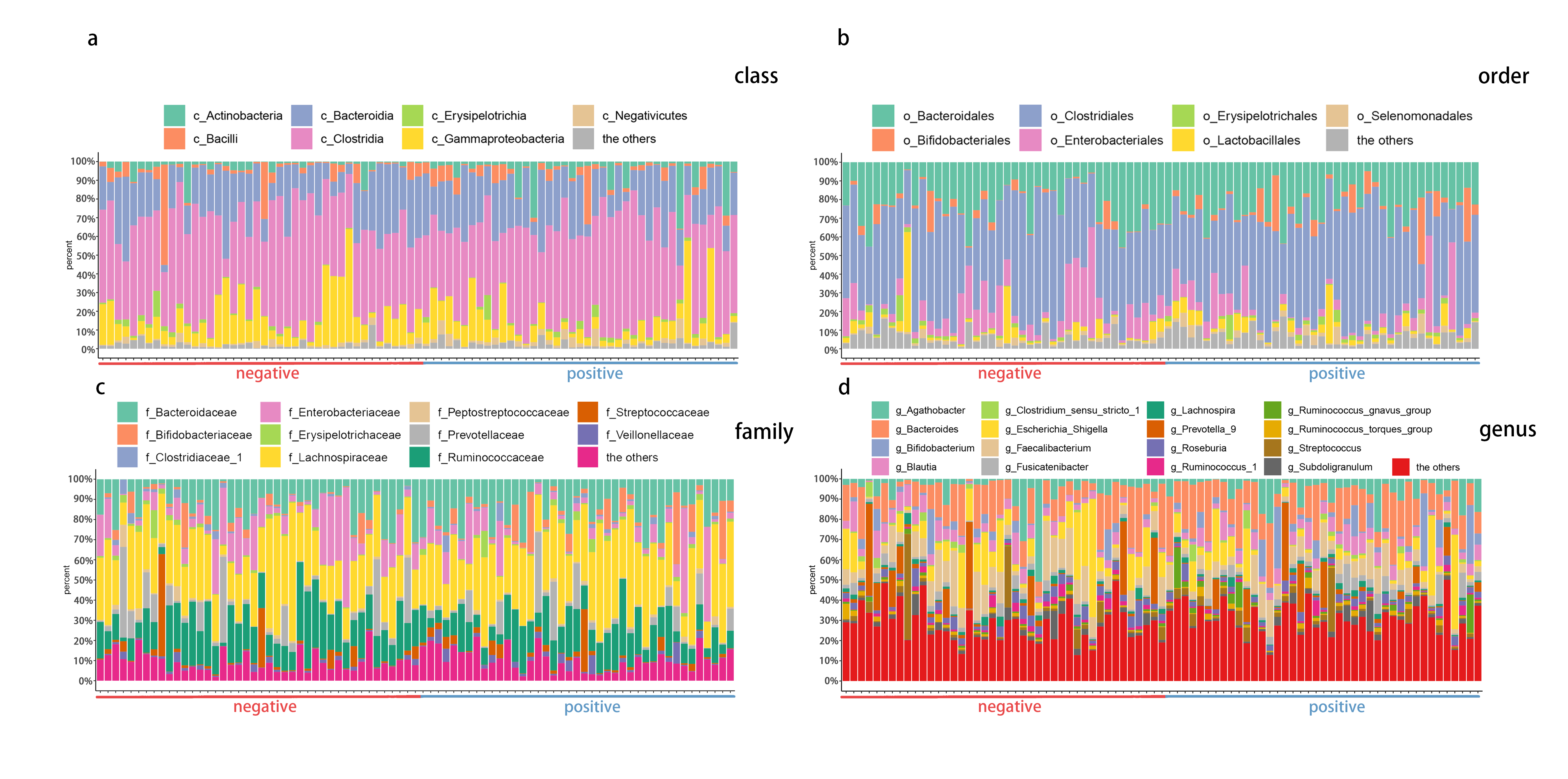

Supplement: Supplementary file 8 — Supplementary Material 8 [file 12866_2023_3132_MOESM8_ESM.png]

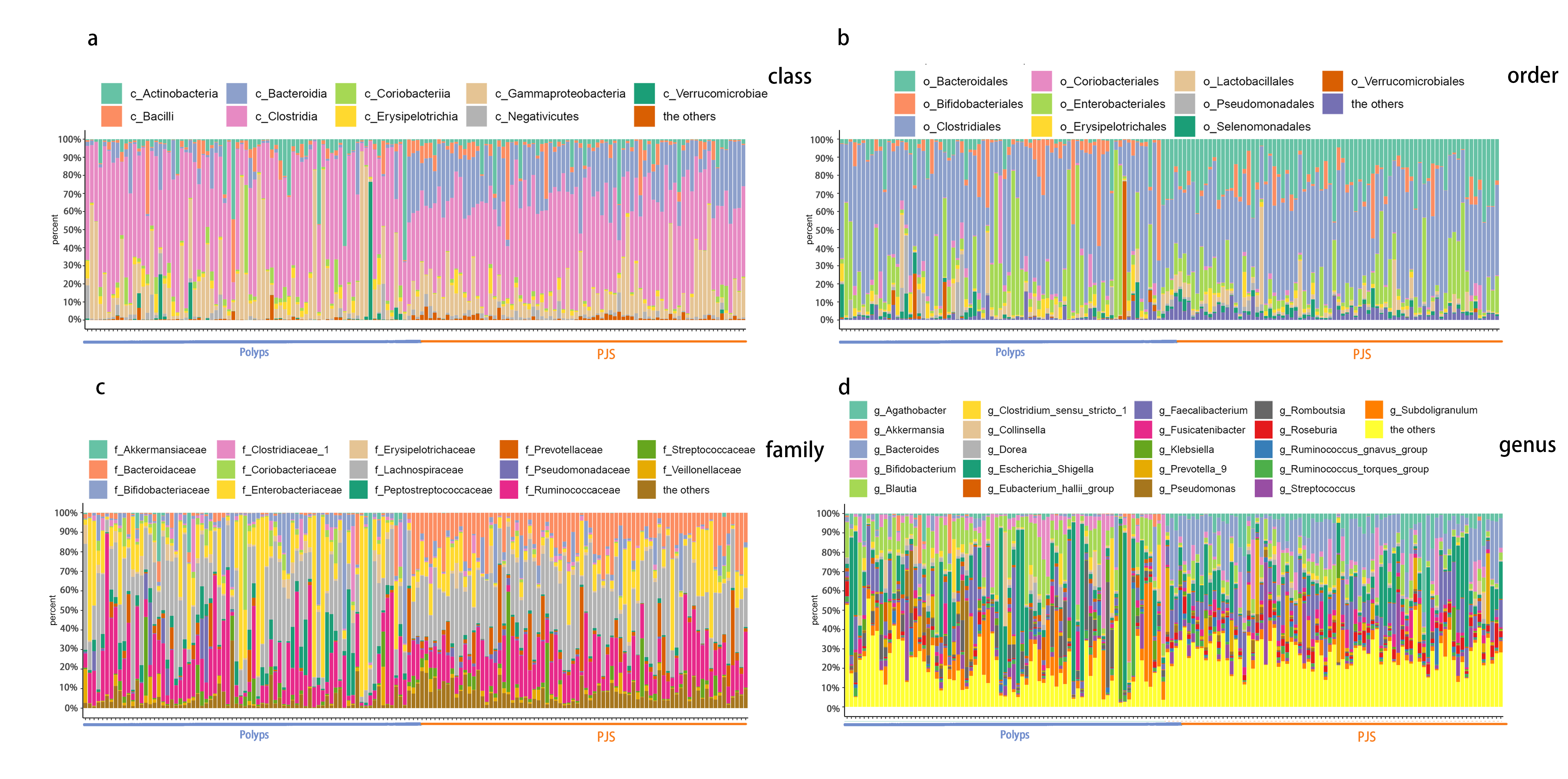

Supplement: Supplementary file 9 — Supplementary Material 9 [file 12866_2023_3132_MOESM9_ESM.png]

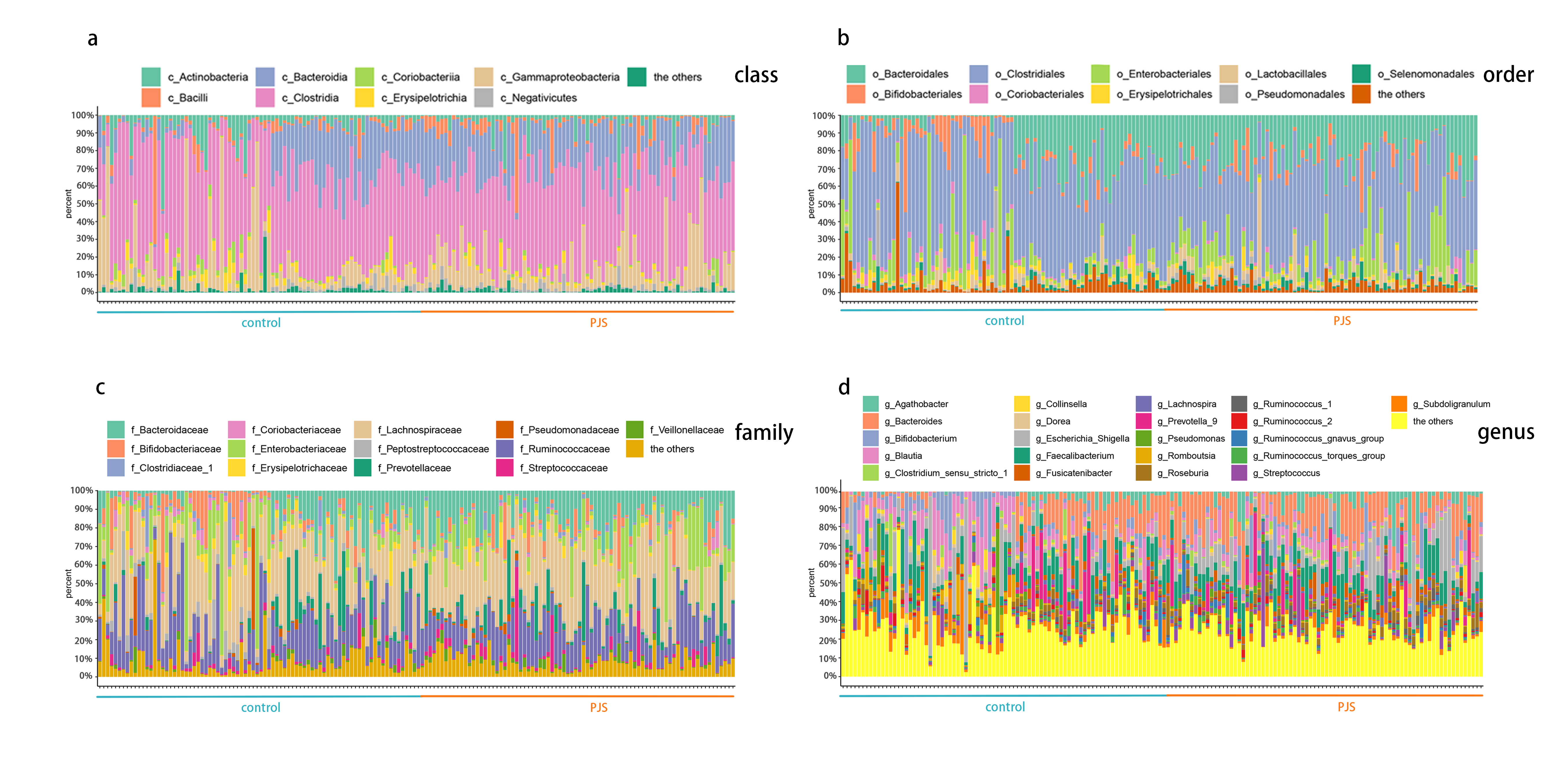

Supplement: Supplementary file 10 — Supplementary Material 10 [file 12866_2023_3132_MOESM10_ESM.png]
