# Supplementary material for: Changes of gut microbiota and short chain fatty acids in patients with Peutz–Jeghers syndrome
Source: BMC Microbiol. 2023 Nov 30;23:373. doi: 10.1186/s12866-023-03132-0 (PMC10688050; doi:10.1186/s12866-023-03132-0)
Supplement: Supplementary file 12 — Supplementary Material 12 [file 12866_2023_3132_MOESM12_ESM.docx]

**Supplements**

**Figure S1** Difference of gut microbiota at the phylum level between PJS patients and healthy controls.

(a) Heatmap of gut microbiota in each sample at the phylum level between PJS patients and healthy controls. (b-d) Difference of Firmicutes, Bacteroidetes and Actinobacteria between PJS patients and healthy controls.

**Figure S2** Difference of gut microbiota at the class, order, family and genus level between PJS patients and healthy controls.

**Figure S3** Boxplot of selected most differentially abundant features at the genus level between PJS patients and healthy controls.

**Figure S4** Difference of gut microbiota at the phylum level between PJS patients and patients with benign polyps.

(a) Heatmap of gut microbiota in each sample at the phylum level between PJS patients and healthy controls. (b-e) Difference of Firmicutes, Bacteroidetes and Actinobacteria between PJS patients and patients with benign polyps.

**Figure S5** Difference of gut microbiota at the class, order, family and genus level between PJS patients and patients with benign polyps.

**Figure S6** Boxplot of selected most differentially abundant features at the genus level between PJS patients and patients with benign polyps.

**Figure S7** Difference of gut microbiota at the phylum level between STK11 positive patients and STK11 negative patients.

(a) Heatmap of gut microbiota in each sample at the phylum level between STK11 positive patients and STK11 negative patients. (b-e) Compairment of Firmicutes, Bacteroidetes, Proteobacteria and Actinobacteria between STK11 positive patients and STK11 negative patients.

**Figure S8** Difference of gut microbiota at the class, order, family and genus level between STK11 positive patients and STK11 negative patients.

**Figure S9** Boxplot of selected most differentially abundant features at the genus level between STK11 positive patients and STK11 negative patients.

**Figure S10** KEGG pathway analysis between PJS patients and healthy controls

1. Top 20 KEGG pathway heat maps (b) Comparison of fatty acid biosynthesis between patients with PJS and healthy controls.

**Figure S11** No significance of the other four SCFAs betweem PJS patients and healthy controls.

**Table S1** Covariance analysis for adjusting the possible confounding factors (age, gender, and BMI) between PJS patients and patients with benign polyps.

**Table S2** The correlation between microbial feature and patient characteristics.

Age: age of onset. Surgery frequency: frequency of endoscopic surgeries. Length: the length of the biggest polyps. Polyps number: number of the PJS polyps.
