# Supplementary material for: Changes of gut microbiota and short chain fatty acids in patients with Peutz–Jeghers syndrome
Source: BMC Microbiol. 2023 Nov 30;23:373. doi: 10.1186/s12866-023-03132-0 (PMC10688050; doi:10.1186/s12866-023-03132-0)
Supplement: Supplementary file 13 — Supplementary Material 13 [file 12866_2023_3132_MOESM13_ESM.docx]

**Table S1** Covariance analysis for adjusting the possible confounding factors (age, gender, and BMI) between PJS patients and patients with benign polyps.

| α-diversity indices | Source | Type III Sumof Squares | df | Mean Square | F | Sig |
| --- | --- | --- | --- | --- | --- | --- |
| Ace | Modified model | 1435535.592 | 4 | 358883.898 | 20.918 | 1.10E-13 |
|  | Intercept | 223763.737 | 1 | 223763.737 | 13.042 | 4.16E-04 |
|  | Group | 746394.364 | 1 | 746394.364 | 43.504 | 7.03E-10 |
|  | Age | 9500.129 | 1 | 9500.129 | 0.554 | 0.458 |
|  | BMI | 926.068 | 1 | 926.068 | 0.054 | 0.817 |
|  | Gender | 6041.324 | 1 | 6041.324 | 0.352 | 0.554 |
| Chao1 | Modified model | 1432709.046 | 4 | 358177.261 | 21.449 | 5.69E-14 |
|  | Intercept | 225092.739 | 1 | 225092.739 | 13.479 | 3.36E-04 |
|  | Group | 739228.601 | 1 | 739228.601 | 44.267 | 5.20E-10 |
|  | Age | 8583.718 | 1 | 8583.718 | 0.514 | 0.475 |
|  | BMI | 956.363 | 1 | 956.363 | 0.057 | 0.811 |
|  | Gender | 6285.964 | 1 | 6285.964 | 0.376 | 0.540 |
| Observed_otus | Modified model | 1346468.555 | 4 | 336617.139 | 23.038 | 8.16E-15 |
|  | Intercept | 216321.524 | 1 | 216321.524 | 14.805 | 1.77E-04 |
|  | Group | 695576.348 | 1 | 695576.348 | 47.605 | 1.42E-10 |
|  | Age | 8936.691 | 1 | 8936.691 | 0.612 | 0.435 |
|  | BMI | 1322.426 | 1 | 1322.426 | 0.091 | 0.764 |
|  | Gender | 6680.319 | 1 | 6680.319 | 0.457 | 0.500 |
| Shannon | Modified model | 78.335^a^ | 4 | 19.584 | 21.656 | 4.41E-14 |
|  | Intercept | 76.197 | 1 | 76.197 | 84.259 | 3.54E-16 |
|  | Group | 28.521 | 1 | 28.521 | 31.539 | 9.39E-08 |
|  | Age | 0.001 | 1 | 0.001 | 0.002 | 0.968 |
|  | BMI | 1.973 | 1 | 1.973 | 2.182 | 0.142 |
|  | Gender | 0.308 | 1 | 0.308 | 0.341 | 0.560 |
