# Supplementary material for: Changes of gut microbiota and short chain fatty acids in patients with Peutz–Jeghers syndrome
Source: BMC Microbiol. 2023 Nov 30;23:373. doi: 10.1186/s12866-023-03132-0 (PMC10688050; doi:10.1186/s12866-023-03132-0)
Supplement: Supplementary file 14 — Supplementary Material 14 [file 12866_2023_3132_MOESM14_ESM.docx]

**Table S2** The correlation between microbial feature and patient characteristics.

Age: age of onset. Surgery frequency: frequency of endoscopic surgeries. Length: the length of the biggest polyps. Polyps number: number of the PJS polyps.

| **Variable** | **Age** | **Surgery frequency** | **Length** | **Polyps number** |
| --- | --- | --- | --- | --- |
| g_Bacteroides | 0.09 (P = 0.43) | 0.1 (P = 0.36) | 0.24 (P = 0.035) | 0.13 (P = 0.26) |
| g_Agathobacter | -0.29 (P = 0.0085) | 0.018 (P = 0.88) | 0. 082 (P = 0.47) | 0.036 (P = 0.75) |
| g_Fusicatenibacter | -0.22 (P = 0.051) | -0.13 (P = 0.25) | -0.012 (P = 0.91) | 0.027 (P = 0.81) |
| g_Roseburia | 0.14 (P = 0.22) | 0.026 (P = 0.82) | -0.13 (P = 0.24) | -0.051 (P = 0.66) |
| g_Escherichia_Shigella | -0.01(P = 0.93) | -0.012 (P = 0.92) | 0.053 (P = 0.64) | 0.1 (P = 0.36) |
| g_Prevotella_9 | 0.0022 (P = 0.98) | 0.12 (P = 0.29) | 0.091 (P = 0.43) | -0.065 (P = 0.57) |
| g_Blautia | 0.051 (P = 0.65) | -0.0065 (P=0.95) | 0.055 (P = 0.63) | -0.11 (P = 0.33) |
| g_Faecalibacterium | 0.0037 (P = 0.97) | -0.17 (P = 0.13) | -0.15 (P = 0.18) | -0.13 (P = 0.26) |
| g_Bifidobacterium | -0.068 (P = 0.55) | 0.0098 (P= 0.93) | -0.2 (P = 0.071) | -0.15 (P = 0.2) |
| g_Subdoligranulum | -0.15 (P = 0.17) | -0.0037 (P=0.97) | -0.19 (P = 0.1) | -0.16 (P = 0.16) |
| Acetic acid | 0.66 (P = 0.015) | -0.39 (P = 0.086) | -0.3 (P = 0.2) | -0.49 (P = 0.027) |
| Propionic acid | 0.57 (P = 0.0091) | -0.44 (P = 0.054) | -0.37 (P = 0.11) | -0.62 (P = 0.036) |
| Butyric acid | 0.65 (P = 0.0021) | -0.47 (P = 0.038) | -0.29 (P = 0.21) | -0.52 (P = 0.018) |
